# The Psychiatric Risk Gene *NT5C2* Regulates Adenosine Monophosphate-Activated Protein Kinase Signaling and Protein Translation in Human Neural Progenitor Cells

**DOI:** 10.1016/j.biopsych.2019.03.977

**Published:** 2019-07-15

**Authors:** Rodrigo R.R. Duarte, Nathaniel D. Bachtel, Marie-Caroline Côtel, Sang H. Lee, Sashika Selvackadunco, Iain A. Watson, Gary A. Hovsepian, Claire Troakes, Gerome D. Breen, Douglas F. Nixon, Robin M. Murray, Nicholas J. Bray, Ioannis Eleftherianos, Anthony C. Vernon, Timothy R. Powell, Deepak P. Srivastava

**Affiliations:** a Social, Genetic & Developmental Psychiatry Centre, Institute of Psychiatry, Psychology & Neuroscience, King’s College London, London, United Kingdom; b Medical Research Council London Neurodegenerative Diseases Brain Bank, Institute of Psychiatry, Psychology & Neuroscience, King’s College London, London, United Kingdom; c Department of Basic & Clinical Neuroscience, Institute of Psychiatry, Psychology & Neuroscience, King’s College London, London, United Kingdom; d Department of Psychosis Studies, Institute of Psychiatry, Psychology & Neuroscience, King’s College London, London, United Kingdom; e Medical Research Council Centre for Neurodevelopmental Disorders, King’s College London, London, United Kingdom; f Medical Research Council Centre for Neuropsychiatric Genetics and Genomics, Cardiff University School of Medicine, Cardiff, United Kingdom; g Department of Biological Sciences, Columbian College of Arts and Sciences, George Washington University, Washington, DC; h Division of Infectious Diseases, Weill Cornell Medicine, Cornell University, New York, New York

**Keywords:** AMP-activated protein kinase (AMPK), *Drosophila melanogaster*, Functional genetics, Neural stem cells, Psychiatric disorders, Ribosomal protein S6 (RPS6)

## Abstract

**Background:**

The 5′-nucleotidase, cytosolic II gene (*NT5C2*, *cN-II*) is associated with disorders characterized by psychiatric and psychomotor disturbances. Common psychiatric risk alleles at the *NT5C2* locus reduce expression of this gene in the fetal and adult brain, but downstream biological risk mechanisms remain elusive.

**Methods:**

Distribution of the NT5C2 protein in the human dorsolateral prefrontal cortex and cortical human neural progenitor cells (hNPCs) was determined using immunostaining, publicly available expression data, and reverse transcriptase quantitative polymerase chain reaction. Phosphorylation quantification of adenosine monophosphate-activated protein kinase (AMPK) alpha (Thr172) and ribosomal protein S6 (Ser235/Ser236) was performed using Western blotting to infer the degree of activation of AMPK signaling and the rate of protein translation. Knockdowns were induced in hNPCs and *Drosophila melanogaster* using RNA interference. Transcriptomic profiling of hNPCs was performed using microarrays, and motility behavior was assessed in flies using the climbing assay.

**Results:**

Expression of *NT5C2* was higher during neurodevelopment and was neuronally enriched in the adult human cortex. Knockdown in hNPCs affected AMPK signaling, a major nutrient-sensing mechanism involved in energy homeostasis, and protein translation. Transcriptional changes implicated in protein translation were observed in knockdown hNPCs, and expression changes to genes related to AMPK signaling and protein translation were confirmed using reverse transcriptase quantitative polymerase chain reaction. The knockdown in Drosophila was associated with drastic climbing impairment.

**Conclusions:**

We provide an extensive neurobiological characterization of the psychiatric risk gene *NT5C2*, describing its previously unknown role in the regulation of AMPK signaling and protein translation in neural stem cells and its association with *Drosophila melanogaster* motility behavior.

The 5′-nucleotidase, cytosolic II gene (*NT5C2, cN-II*) encodes a phosphatase associated with disorders characterized by psychiatric and psychomotor disturbances, including schizophrenia 1, 2, 3, 4, Parkinson’s disease (5), and spastic paraplegia (6). The NT5C2 enzyme cleaves purinergic monophosphate nucleotides and has a particularly high affinity for adenosine monophosphate (AMP) (7). These energetic molecules are required for the extensive transcriptional programming governing cell maintenance, proliferation, migration, and differentiation during neurodevelopment 8, 9, 10, 11 and have been previously implicated in adult brain function and psychiatric and psychomotor disturbances 12, 13, 14.

We previously showed that common psychiatric risk variants at the *NT5C2* locus are associated with reduced neurological expression of this gene in population control subjects and in the fetus (3). As a key regulator of intracellular AMP, we hypothesize that NT5C2 modulates the AMP-activated protein kinase (AMPK), a major energy homeostasis regulator 15, 16. AMPK signaling has been previously associated with psychiatric disorders 17, 18, 19, 20, *NT5C2* function in muscle fibers (21), and highly energy consuming processes such as protein translation 22, 23, 24, 25, 26, 27 and motility behavior 17, 28, 29. However, the underlying gene regulatory networks that mediate the effect of *NT5C2* on AMPK signaling in the context of psychiatric disorders, and the relevant cell types and developmental time points, remain unclear.

In this study, we investigated *NT5C2* expression, function, and protein distribution in the human brain and human neural progenitor cells (hNPCs); its role in the regulation of AMPK signaling and protein translation; and its association with climbing behavior in *Drosophila melanogaster*. First, to extend our previous work, we identified the major cell types expressing *NT5C2* in the adult brain, which showed that NT5C2 protein is more expressed in neurons relative to glial cells. Second, we gathered complementary evidence that this gene is more expressed and therefore likely to play a functional role during neurodevelopment. Third, we investigated the effects of *NT5C2* loss-of-function on the phosphorylation of AMPK alpha (Thr172) and ribosomal protein S6 (RPS6) (Ser235/Ser236) in hNPCs, suggesting a regulatory role in AMPK signaling and protein translation. Finally, based on evidence from genetic studies implicating *NT5C2* in psychomotor disturbances, we tested the effect of a loss-of-function on climbing behavior using *D. melanogaster* as model organism, confirming a role in motility behavior. The present study provides an extensive neurobiological characterization of *NT5C2*, describing its hitherto unknown relationship with AMPK signaling and protein translation in neural stem cells and a role in motility behavior in the fly. Ultimately, these data demonstrate biological mechanisms associated with *NT5C2* that may explain its association with psychiatric disorders.

## Methods and Materials

See Supplemental Methods and Materials for further details.

### Brain Samples

To identify cell type–specific expression of *NT5C2* in the adult cortex, we obtained samples from unaffected control subjects from the Medical Research Council London Neurodegenerative Disease Brain Bank, Institute of Psychiatry, Psychology & Neuroscience, King’s College London (UK Human Tissue Authority license #12293).

### Immunohistochemistry and Cytochemistry

Brain sections were deparaffinized and submitted to antigen retrieval and autofluorescence removal protocols (Supplemental Methods and Materials). hNPCs were fixed and processed as previously described (30). The following primary antibodies were used: NT5C2 monoclonal antibody (M02-3C1) (Abnova, Taipei, Taiwan), ionized calcium-binding adapter molecule 1 (IBA1) (Menarini Diagnostics Ltd., Winnersh, United Kingdom), glial fibrillary acidic protein (GFAP) (Agilent, Santa Clara, CA), microtubule-associated protein 2 (MAP2) (Abcam, Cambridge, United Kingdom), parvalbumin (PARVALB) (Abcam), and beta III tubulin (Abcam). Fluorescently labeled secondary antibodies included goat or rabbit Alexa 488, 568, and 633 antibodies (Thermo Fisher Scientific, Waltham, MA).

### Cell Lines

We used hNPCs from the CTX0E16 neural stem cell line (31) or from human induced pluripotent stem cells from an unaffected control subject (30) and human embryonic kidney 293T (HEK293T) cells to identify the subcellular distribution and function of *NT5C2*. The CTX0E16 neural cell line (31) was obtained from ReNeuron Ltd. (Bridgend, United Kingdom) under a Material Transfer Agreement. All lines were derived and maintained as described in the Supplemental Methods and Materials and elsewhere 30, 31.

### RNA and Protein Isolation and Quantification

To identify gene and protein expression and phosphorylation differences in knockdown or overexpression cultures, we isolated total RNA or protein using TRI Reagent or RIPA Buffer supplemented with Halt Protease and Phosphatase Inhibitor Cocktail (Thermo Fisher Scientific), respectively. Reverse transcriptase quantitative polymerase chain reaction (RT-qPCR), RNA quality control, and Western blotting details are available in the Supplemental Methods and Materials. Primary antibodies for Western blotting included AMPK alpha (D6) and phospho-AMPK alpha (Thr172) (Santa Cruz Biotechnology, Dallas, TX) and total RPS6 (54d2) and phospho-RPS6 (Ser235/Ser236) (Cell Signaling Technology, Danvers, MA).

### Fly Stocks and Climbing Assay

We used Gal4-upstream activated sequences (UAS) (32) to knock down *CG32549* in specific tissues by crossing a *CG32549*-RNA interference (RNAi line) (knockdown: v30079) with UAS lines where Gal4 expression (and thus knockdown) is driven throughout the body (*ACT5C*: BL4414), in neurons and neural progenitor cells (*ELAV*: BL8765), or in gut (*GUT*: DGRC113094). Crosses were submitted to the negative geotaxis assay (33), a cost-effective test that has been previously used to investigate the association between genes and motility 34, 35, 36. Climbing success was calculated as percentage of flies per tube that climbed over an arbitrary mark, and survival was determined as percentage of flies alive 17 to 20 days after emergence, out of starting flasks containing 20 flies (*n* = 4+ flasks per condition).

### Statistical Analysis

To infer statistical differences between more than two independent groups, we used one-way analysis of variances followed by Tukey post hoc tests (for normally distributed values) or Kruskal-Wallis tests followed by Dunn’s tests (for non–normally distributed values). To infer differences between two groups, we performed two-way independent parametric *t* tests. Multiple testing correction was applied as described in Results. Microarray expression data were analyzed using linear regressions (Supplemental Methods and Materials). Gene overlap significance was calculated in R (R Foundation for Statistical Computing, Vienna, Austria; https://www.R-project.org) using Fisher’s exact test (GeneOverlap package). Statistical analyses were performed in R and SPSS version 24 (IBM Corp., Armonk, NY).

## Results

### Expression of NT5C2 Is Enriched in Neurons in the Adult Brain

To extend our previous work and investigate the relationship between *NT5C2* expression and psychiatric disorders, we investigated which cell types express this gene in the adult brain. We examined single-cell RNA-sequencing data from the mouse cortex (37), which revealed cell type–specific profiles of *NT5C2* expression (Kruskal-Wallis test, H_4_ = 52.44, *p* < .001). Post hoc analyses confirmed that *NT5C2* was more frequently observed in pyramidal neurons (95th percentile = 3 [counts]) than astrocytes (95th percentile = 1, Dunn’s post hoc test, *p*_*corrected*_ < .001) and in interneurons (95th percentile = 3) than microglia (95th percentile = 2.05, *p*_*corrected*_ < .01) or astrocytes (95th percentile = 1, *p*_*corrected*_ < .001) (Supplemental Figure S1).

**Figure 1:**
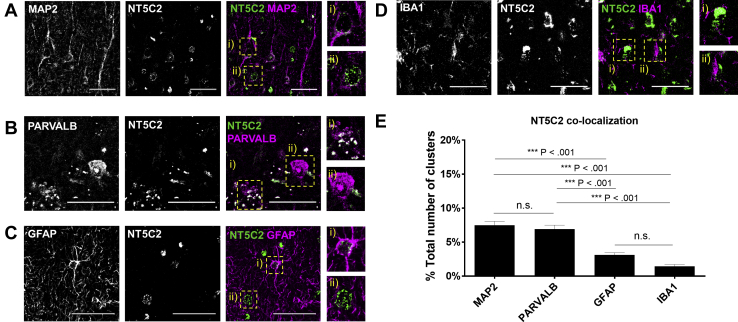
Distribution of the psychiatric risk protein NT5C2 in dorsolateral prefrontal cortex sections of postmortem unaffected individuals. Colocalization of NT5C2 staining with **(A)** microtubule-associated protein 2 (MAP2) (neuronal marker), **(B)** parvalbumin (PARVALB) (interneuron marker), **(C)** glial fibrillary acidic protein (GFAP) (glial marker), and **(D)** ionized calcium-binding adapter molecule 1 (IBA1) (microglia marker). Scale bars = 50 μm. **(E)** Quantification of the colocalization of NT5C2 with markers from panels **(A–D)** revealed a cell type–specific expression profile of NT5C2 in the mature cortex (one-way analysis of variance, Tukey pairwise comparisons: ***p < .001 for all comparisons). n.s., not significant.

To investigate correlates with the human cortex, we performed a series of immunocolocalization experiments using postmortem brains. Initially, we investigated the specificity of an antibody for NT5C2 by probing HEK293T cells and CTX0E16 hNPCs overexpressing myc-NT5C2 (Supplemental Figures S2, S4). While immunolabeling of endogenous expression in heterologous systems may produce different results owing to the existence of tissue-specific isoforms, these findings corroborate the suitability of this antibody for our aims. This antibody was used to investigate the distribution of NT5C2 in the prefrontal cortex using standard immunoperoxidase staining with 3,3′-diaminobenzidine as chromogen (Supplemental Figure S3). Preliminary analysis of NT5C2 immunostaining using Nissl counterstain (to reveal cell morphology) suggested that NT5C2 was present in neurons, glia, and the surrounding neuropil. However, we noted that not all putative glial cells expressed NT5C2 (red arrows in Supplemental Figure S3), corroborating a previous observation by the Human Protein Atlas that expression is lower in these cells (38). To confirm this, we investigated the cell type–specific expression profile of NT5C2 in the cortex by quantifying immunocolocalization of this protein with markers of mature neurons (MAP2), a subclass of gamma-aminobutyric acid interneurons (PARVALB), astrocytes (GFAP), and microglia (IBA1) (Figure 1A–E). These cells were selected based on the wealth of evidence implicating them in the pathophysiology of psychiatric disorders 39, 40, 41, 42. Colocalization quantification was performed using a semiautomated ImageJ (National Institute of Mental Health, Bethesda, MD) macro 43, 44 (Supplemental Methods and Materials), which revealed cell type–specific profiles of NT5C2 expression (one-way analysis of variance, *F*_3,44_ = 39.12, *p* < .001, *n* = 4 unaffected control individuals, 4 technical replicates each, 20 fields of view per technical replicate). Colocalization occurred more frequently with neuron and interneuron markers than nonneuronal markers (Tukey post hoc tests: MAP2 [7.5% (cluster colocalization relative to all clusters in image) ± 2.0% (SD)], PARVALB [6.9% ± 2.1%], GFAP [3.13% ± 1.1%], IBA1 [1.4% ± 0.93%]; *p* < .001 for all comparisons except MAP2 vs. PARVALB and GFAP vs. IBA1 [*p* > .05]) (Figure 1E). NT5C2 expression at the transcript and protein levels appeared to be more highly expressed in neurons within the adult brain, consistent with recent observations that risk variants implicated in schizophrenia are enriched for neuronal genes (40).

### NT5C2 Is Highly Expressed and Ubiquitously Distributed in hNPCs

**Figure 2:**
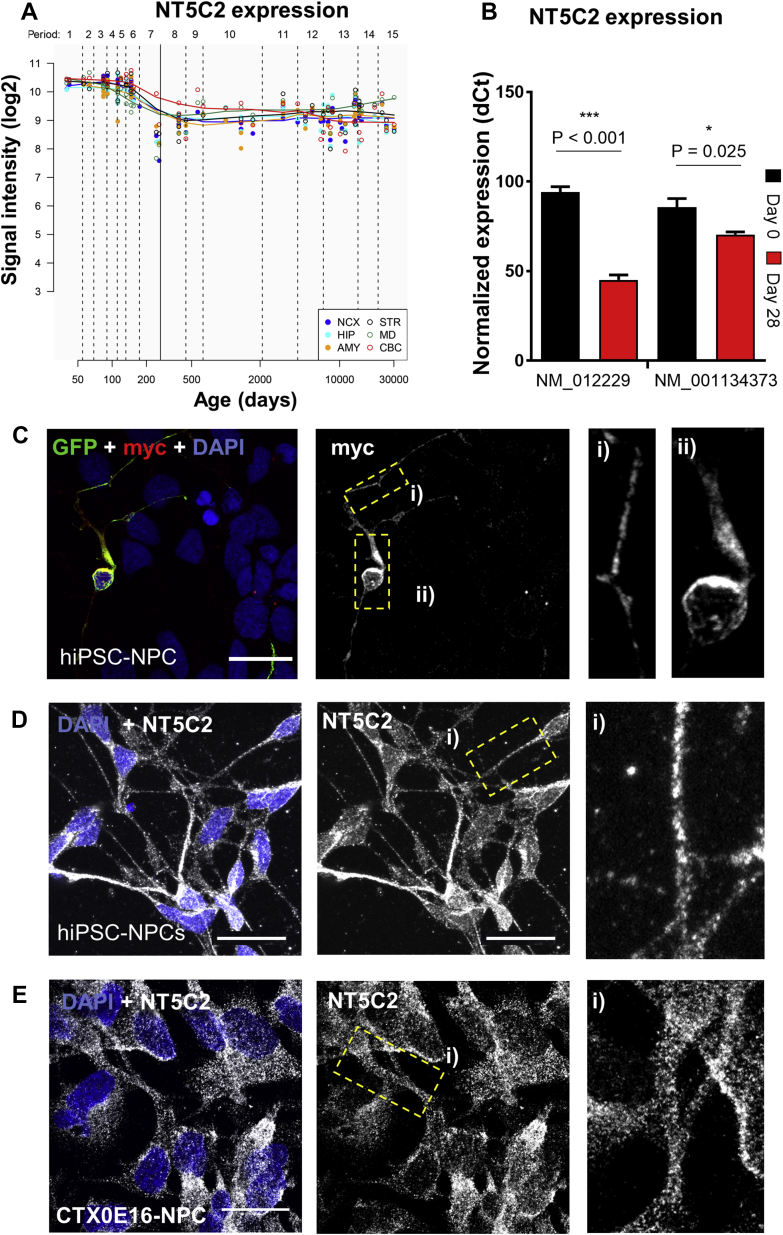
Neurodevelopmental expression of *NT5C2* and protein distribution in human neural progenitor cells (hNPCs). **(A)** Neurological expression of *NT5C2* across human development, according to the Human Brain Transcriptome Atlas (46), showing that expression peaks during fetal development. **(B)** Expression of *NT5C2* RefSeq transcripts NM_012229 and NM_001134373 in hNPCs (day 0) and cultures differentiated for 28 days. Expression is significantly higher at the neural progenitor state. *t* tests: ***p < .001, *p < .05. **(C)** Distribution of ectopic NT5C2 was assessed by transfecting hNPCs with plasmids encoding NT5C2-myc and enhanced green fluorescent protein (GFP), followed by immunolabeling using antibodies raised against myc or GFP (GFP was used as morphological marker). **(D)** Subcellular localization of endogenous NT5C2 in hNPCs derived from a human induced pluripotent stem cell (hiPSC) line and from **(E)** the CTX0E16 cell line. NT5C2 was ubiquitously distributed in hNPCs from both models. Scale bars = 20 μm. AMY, amygdala; CBC, cerebellar cortex; dCt, delta cycle threshold; HIP, hippocampus; MD, mediodorsal nucleus of the thalamus; NCX, neocortex; STR, striatum.

The role of *NT5C2* in psychiatric disorders has been previously hypothesized to begin during neurodevelopment, a period underscored by complex processes implicated in major psychiatric disorders (45), with this risk mechanism persisting in the adult brain (3). The contribution of *NT5C2* to neurobiology, however, should be greater at the developmental stage when this gene is more expressed. Thus, we investigated *NT5C2* expression in the Human Brain Transcriptome atlas (46), which revealed that expression peaks during neurodevelopment (Figure 2A). Considering that established cell lines are cost-effective and easy-to-use tools to study neurodevelopment, we tested whether the CTX0E16 hNPC line 30, 31 would constitute an appropriate model. We measured expression of the *NT5C2* main RefSeq transcripts (NM_012229 and NM_001134373) in these cells, specifically in hNPCs and immature neuronal cultures terminally differentiated for 28 days, which we previously showed to comprise neurons (approximately 80%) and glial cells (approximately 10%) 30, 31. The expression of *NT5C2* RefSeq transcripts NM_012229 and NM_001134373 in hNPCs (day 0; NM_012229: 94.22 ± 5.85; NM_001134373: 85.67 ± 9.77) was 30% higher compared with neuronal cultures terminally differentiated for 28 days (NM_012229: 45.09 ± 5.59; NM_001134373: 70.45 ± 2.93; *t* tests: NM_012229, *t*_6_ = 12.14, *p* < .001, Bonferroni corrected *p* < .001; NM_001134373, *t*_6_ = 2.99, *p* = .0245, Bonferroni corrected *p* = .049). These findings are consistent with higher expression of *NT5C2* at an earlier developmental stage, with persistent expression after differentiation (Figure 2B).

As subcellular protein distribution can inform gene function, we immunolabeled hNPCs from the CTX0E16 and human induced pluripotent stem cell lines to study NT5C2 localization. We ectopically expressed a myc-tagged NT5C2 construct in human induced pluripotent stem cell NPCs and labeled these cells using myc or NT5C2 antibodies, which revealed that myc-NT5C2 was abundantly expressed in punctate structures in the cell soma and along neurites (Figure 2C; Supplemental Figure S4). Similarly, endogenous NT5C2 was distributed in punctate structures through the cell and neurites (Figure 2D, E), suggesting that this protein is ubiquitously distributed in hNPCs, consistent with the expected distribution of a cytosolic protein.

### NT5C2 Regulates the Phosphorylation of AMPK and RPS6

**Figure 3:**
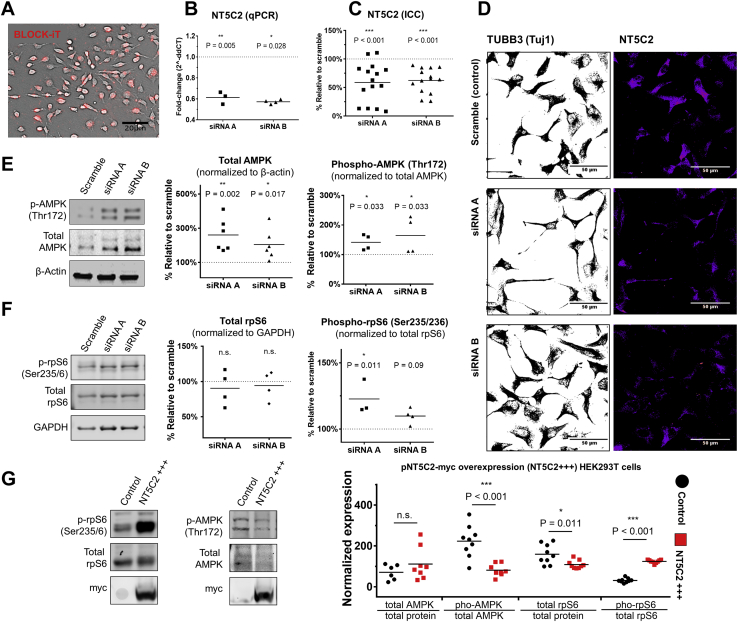
Knockdown of *NT5C2* in human neural progenitor cells is associated with differential phosphorylation of adenosine monophostate-activated protein kinase (AMPK) and ribosomal protein S6 (RPS6). **(A)** The efficiency of the small interfering RNA (siRNA) transfection was determined by uptake of BLOCK-iT, a fluorescently labeled oligonucleotide. **(B)***NT5C2* expression was significantly reduced in knockdown cultures (linear regressions covarying for biological replicates, Tukey post hoc tests, **p < .01, *p < .05). **(C, D)** siRNA treatments significantly reduce *NT5C2* expression in independent human neural progenitor cell cultures at the protein level (one-way analysis of variance, Tukey post hoc tests, ***p < .001). **(E)** The *NT5C2* knockdown was associated with increased total AMPK alpha and phosphorylated AMPK alpha (pAMPK) (Thr172) in human neural progenitor cells (Kruskal-Wallis test, Dunn’s post hoc tests, **p < .01, *p < .05). **(F)** The knockdown did not alter total RPS6 levels but was associated with increased phosphorylated RPS6 (prpS6) (Ser235/Ser236). siRNA A was associated with a mean 23% increase in phosphorylation (Kruskal-Wallis test, Dunn’s test, *p < .05), and siRNA B was associated with a modest 10% mean increase, which was not significant after correction (*p* = .09). Full blots for panels (**E** and **F**) are available in Supplemental Figure S8. **(G)** The overexpression of *NT5C2* in human embryonic kidney 293T (HEK293T) cells causes a significant decrease in phosphorylated AMPK alpha levels and in total RPS6, and a significant increase in phosphorylated RPS6 (*t* test, ***p < .001, *p < .05). Full blots are available in Supplemental Figure S9. ddCt, delta-delta cycle threshold; ICC, immunocytochemistry; n.s., not significant; qPCR, quantitative polymerase chain reaction.

The knockdown of *NT5C2* activates AMPK signaling in muscle fibers (21), and considering the relevance of AMPK to psychiatry 17, 18, 19, 20, we tested whether this also occurred in hNPCs. The knockdown in CTX0E16 hNPCs was performed using two independent small interfering RNA (siRNA) sequences, A and B. Initially, the transfection protocol efficacy was determined by assessing the uptake of fluorescently labeled oligonucleotides (BLOCK-iT; Thermo Fisher Scientific), which revealed a transfection rate of 90% ± 0.02% (*n* = 4 biological replicates per condition, 4 technical replicates each) (Figure 3A). We obtained knockdown cultures and confirmed reduced *NT5C2* expression using RT-qPCR (linear regression to identify the effect of siRNAs on *NT5C2* expression controlling for biological replicate: *F*_2,5_ = 13.9, *p* = .009, *R*^*2*^ = .92; Tukey post hoc tests against scramble [3.29 (delta cycle threshold) ± 0.23 (SD)]: siRNA A [3.79 ± 0.09], fold change = 0.71, *p* = .005; siRNA B [3.29 ± 0.23], fold change = 0.81, *p* = .028) (Figure 3B). The ability of these siRNAs to knockdown NT5C2 protein was further assessed in independent cultures (Figure 3C, D), which revealed a significant decrease in protein abundance in knockdown conditions (one-way analysis of variance, *F*_2,41_ = 12.23, *p* < .001; Tukey post hoc tests against scramble [100.0 ± 14.7]: siRNA A [58.8 ± 34.7], *p* < .001; siRNA B [62.4 ± 21.5], *p* < .001).

To test the effect of the knockdown on AMPK signaling, we measured total abundance and relative phosphorylation of AMPK alpha, a subunit of AMPK. We observed a significant effect of the knockdown on AMPK alpha abundance, which was associated with a mean 132% increase in total protein (Kruskal-Wallis test, H_3_ = 12.2, *p* < .001; Dunn’s post hoc tests against scramble [median (Mdn) = 100.0]: siRNA A [Mdn = 236.1], *p* = .002; siRNA B [Mdn = 182.8], *p* = .017) (Figure 3E). Additionally, we observed a significant effect of the knockdown on the relative phosphorylation of AMPK alpha (Thr172), with the knockdown associated with a mean 55% increase in phosphorylated AMPK, suggesting activation of this cascade (Kruskal-Wallis test, H_3_ = 7.65, *p* < .013; Dunn’s post hoc tests against scramble [Mdn = 100.0]: siRNA A [Mdn = 141.2], *p* = .033; siRNA B [Mdn = 160.7], *p* = .033) (Figure 3E).

Considering that protein translation is partly regulated by AMPK (23) and is one of the most energy-consuming cellular processes (47), we hypothesized that *NT5C2* function could affect the rate of protein synthesis. To test this, we assessed the effects of the knockdown on the phosphorylation of RPS6 (Ser235/Ser236), which is routinely used as a proxy to estimate the rate of protein translation in neurons, as it correlates with mammalian target of rapamycin complex 1 activation (48). No difference was observed in total RPS6 abundance (Kruskal-Wallis test, *p* > .05) (Figure 3F), but we observed that the knockdown with siRNA A was associated with a mean 23% increase in phosphorylated RPS6 (Kruskal-Wallis test, H_3_ = 8.22, *p* = .002; Dunn’s post hoc test against scramble [Mdn = 100.0]: siRNA A, Mdn = 115.9, *p* = .012) (Figure 3F). The knockdown with siRNA B elicited a mean 10% increase in RPS6 phosphorylation, but this did not survive correction (siRNA B, Mdn = 110.2, *p* = .09).

We obtained complementary evidence supporting the association between *NT5C2* and AMPK and RPS6 regulation using HEK293T cells. Overexpression of ectopic myc-tagged NT5C2 (NT5C2-myc) in these cells resulted in a mean 64% decrease in phosphorylated AMPK alpha (*t* test, control [no vector]: 223.00 [normalized expression] ± 76.99 [SD], overexpression: 81.05 ± 30.14, *t*_15_ = 4.88, *p* < .001, Bonferroni-adjusted [four tests] *p* < .001), whereas no difference in total AMPK levels was observed (*p* > .05) (Figure 3G). These results are consistent with our previous data and indicate that NT5C2 is a negative regulator of AMPK signaling. Subsequently, we observed a mean 28% decrease in total RPS6 abundance (*t* test, control: 159.10 ± 48.52, overexpression: 108.8 ± 48.52, *t*_16_ = 2.88, *p* = .011, *p*_*corrected*_ = .044) and a highly significant 300% increase in RPS6 phosphorylation (*t* test, control: 31.03 ± 10.66, overexpression: 124.10 ± 8.20, *t*_16_ = 20.76, *p* < .001, *p*_*corrected*_ < .001) (Figure 3G). The effect of exogenous *NT5C2* on RPS6 here was opposite to what we observed in hNPCs, highlighting the complex nature of the intracellular cascades governing protein translation, which are examined in the Discussion.

### NT5C2 Is Associated With Transcriptional Changes Implicated in Protein Translation

**Figure 4:**
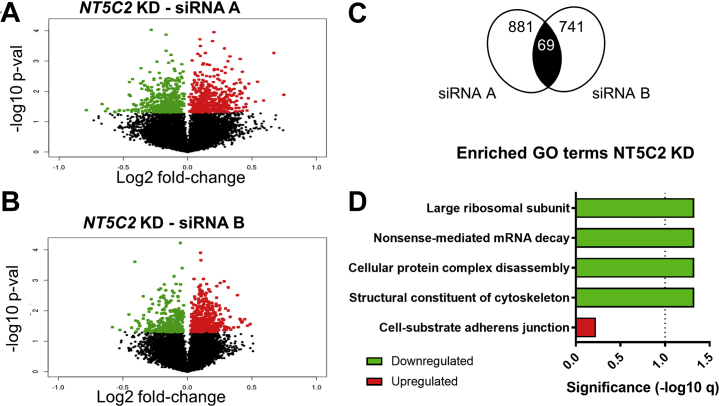
Transcriptional changes associated with the knockdown (KD) corroborate a role for *NT5C2* on protein translation. Volcano plots indicate nominally significant transcriptomic changes elicited by **(A)** small interfering RNA (siRNA) A and **(B)** siRNA B. **(C)** Venn diagrams indicating the number of genes differentially regulated by siRNA A and siRNA B and the overlapping gene set, which is unlikely to occur by chance, according to Fisher’s exact test (*p* < .001). **(D)** Gene ontology (GO) terms enriched within genes concordantly, differentially expressed in both KD conditions. The line indicates the significance threshold (−log10 *q* < .05). mRNA, messenger RNA; p-val, *p* value.

To determine the regulatory gene networks governing the effect of *NT5C2* on AMPK signaling in hNPCs, we profiled the transcriptome of these cultures using microarrays (Figure 4A, B). We aimed to characterize expression differences that were shared between both siRNA treatments to reduce off-target effects associated with individual siRNAs (49). The concordant transcriptomic changes consisted of an overlap of 69 genes (Figure 4C), which is statistically unlikely to occur by chance (genes in microarray = 21,196; affected by siRNA A: 881 genes; siRNA B: 741 genes; Fisher’s exact test, *p* < .001, Jaccard index < 0.001, odds ratio = 2.6) (gene list in Supplemental Table S1). We observed that there was a high correlation between samples within the same siRNA groups, despite the modest number of overlapping, differentially expressed genes (Pearson’s *r* > .99, *p* < .001 for all correlations; siRNA A, *n* = 3 biological replicates; siRNA B and scramble, *n* = 4). The list of overlapping gene expression changes was subdivided by directionality of effect, and the upregulated and downregulated gene network topologies were constructed using GeneMANIA (50) (Supplemental Figure S5). This analysis revealed multiple connections between genes owing to coexpression and colocalization, corroborating their functional association. The upregulated and downregulated gene lists were analyzed for enrichment of gene ontology terms (Figure 4D; Supplemental Table S2), which revealed downregulated genes (*q* < .05) pertaining to the regulation of protein translation, and of the cytoskeleton [which is highly dependent on the transcriptional machinery (51)]. Furthermore, ribosomal genes, including *RPL15*, *RPL22*, *RPL5*, and *RPL6*, were downregulated, consistent with activation of AMPK signaling and inhibition of protein translation. The top upregulated gene ontology term suggested the involvement of *NT5C2* in cell adhesion, but this was not significant after correction (*q* > .05).

We used RT-qPCR to validate a panel of gene expression changes observed in the microarray analysis (Supplemental Figure S6), which were related to protein translation and AMPK signaling, including the heterogeneous nuclear ribonucleoprotein A1 (*HNRNPA1*), the proteasome 26S subunit, ATPase 4 (*PSMC4*), and the autophagy-related cysteine peptidase gene (*ATG4B*). *HNRNPA1* is involved in protein translation (52), whereas *ATG4B* regulates AMPK signaling and energy homeostasis (53), and *PSMC4* physically interacts with AMPK (54) and is involved in Parkinson’s disease (55). Considering the effects of NT5C2 in AMPK and RPS6 regulation, the transcriptional changes observed here corroborate a role for *NT5C2* in protein translation, providing evidence of the gene networks involved.

### Knockdown of *CG32549* in *D. melanogaster* Is Associated With Impaired Climbing

**Figure 5:**
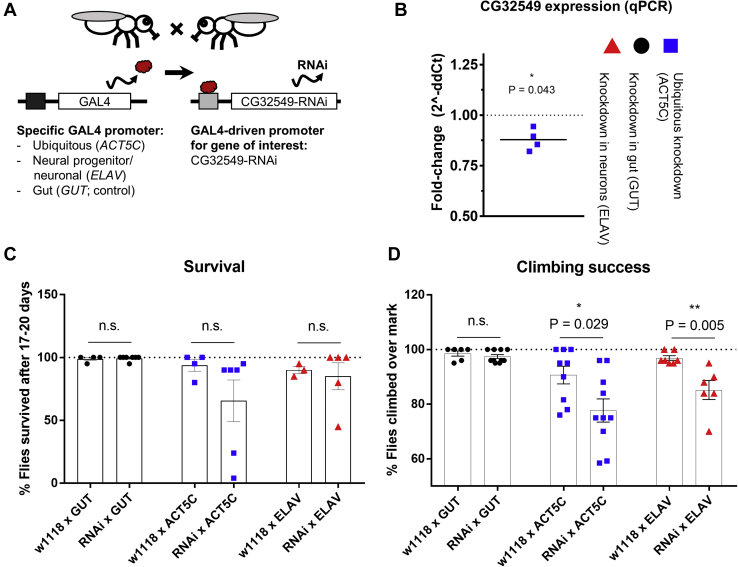
Knockdown of *CG32549* (*NT5C2* homologue) in *Drosophila melanogaster* using the Gal4-upstream activated sequences (UAS) system. *CG32549*-RNA interference (RNAi) was induced ubiquitously (*ACT5C* promoter), in gut (*GUT*), or in neural progenitor cells and neurons (*ELAV*). **(A)** Experimental design of the knockdown. **(B)***CG32549* was less expressed in the brain of knockdown flies (*t* test, *p < .05). **(C)** There was no difference in survival percentage between UAS lines and UAS-knockdown lines 17–20 days after emergence (*t* tests, *p* > .05). **(D)** Climbing success observed in UAS lines vs. UAS-knockdown lines highlight the effect of the ubiquitous and neuron-specific knockdowns on motility (*t* tests, *p < .05, **p < .01), an effect that was not observed in the gut-specific condition (*p* > .05). ddCt, delta-delta cycle threshold; n.s., not significant; qPCR, quantitative polymerase chain reaction.

Considering the genetic link between *NT5C2* and disorders associated with psychiatric and psychomotor disturbances and the importance of AMPK in energy-consuming tasks such as motility 19, 56, we investigated a potential role of the *NT5C2* homologue of Drosophila in climbing. The human NT5C2 protein shares 60.5% sequence identity and 80.2% sequence similarity with CG32549 (Supplemental Figure S7), suggesting that these proteins exert the same or similar function. To investigate the role of CG32549 in motility while controlling for potential confounding effects in muscles, we generated flies using the Gal4-UAS system to obtain crosses with reduced expression of this gene ubiquitously (driven by *ACT5C* promoter), in neurons and neural progenitor cells (*ELAV*), or in gut as a control (*GUT*) (Figure 5A). The ubiquitous knockdown was associated with reduced *CG32549* expression in the brain (UAS line [control, no RNAi cassette] = 0.042 [delta cycle threshold] ± 0.027; UAS-KD [knockdown] flies = 0.007 ± 0.004; fold change = 0.88; *t* test: *t*_6_ = 2.6, *p* = .043, *n* = 4) (Figure 5B). No difference in survival was observed across genotypes (UAS vs. UAS-KD lines, *t* tests, *p* > .05, *n* = 5 flasks on average) (Figure 5C). We observed a significant impairment in climbing success associated with the knockdown using the *ELAV* promoter (UAS = 96.9% ± 2.2%, UAS-KD = 85.2% ± 8.5%; *t*_11_ = 3.53, *p* = .005, adjusted *p* = .014, *n* = 7 per condition on average) (Figure 5D). There was also a nominally significant reduction in climbing success on knockdown of *CG32549* using the *ACT5C* promoter (UAS = 90.6% ± 9.7%, UAS-KD = 77.7% ± 13.4%, *t* test: *t*_17_ = 2.4, *p* = .029, *n* = 10 per condition on average, Bonferroni (adjusted for three comparisons) *p* > .05). This impairment was not observed in flies with the knockdown in gut (UAS = 98.5% ± 2.3%, UAS-KD = 97.4% ± 2.3%, *t* test, *p* > .05, *n* = 8 per condition on average). These findings suggest there is an effect of neuronal *CG32549* in *D. melanogaster* motility and provide an insight into the function of *NT5C2* at a systems level*.*

## Discussion

The *NT5C2* gene is implicated in risk for psychiatric and neurological conditions 1, 2, 3, 5, 6, and it has been recently classified as a high confidence schizophrenia risk gene by PsychENCODE (4), but the biological mechanisms responsible for these associations remain elusive. We previously showed that psychiatric risk alleles at the *NT5C2* locus are associated with reduced expression of this gene in the adult and developing brain (3). In this study, we observe that reduced *NT5C2* expression is associated with differential regulation of AMPK signaling and RPS6 in hNPCs, suggesting a regulatory role in energy homeostasis and protein translation. We also observe that neurological expression of *NT5C2* peaks during neurodevelopment but persists at later developmental stages, corroborating our previous hypothesis that the *NT5C2* risk mechanism is an ongoing process that starts from embryonic development (3). The cell type–specific *NT5C2* expression profile observed in the adult brain further revealed an enrichment for neuronal expression, suggesting that reduced *NT5C2* expression in the mature cortex could be particularly detrimental to neurons. These findings are consistent with recent studies showing that schizophrenia risk variants are enriched for genes implicated in neurodevelopment and neuronal function 40, 57.

The manipulation of *NT5C2* expression in hNPCs and HEK293T cells corroborates the existence of a complex regulatory network governing protein translation, with observations suggesting, at first glance, opposing effects of AMPK on the rate of protein synthesis. However, on closer inspection, we observed that our findings with HEK cells corroborate that AMPK activation inhibits protein translation by repressing the mammalian target of rapamycin complex 1 and the eukaryotic translation elongation factor 2 23, 24, 58, 59. Our findings with the hNPC model, in turn, corroborate AMPK activation leading to increased rates of protein synthesis over time, despite an initial period in which translation is halted, likely owing to a negative feedback loop (60). As a result, we observed increased abundance of AMPK alpha in the neural progenitor cell cultures, whereas no differences in expression of AMPK transcripts were detected.

We also observed that a knockdown of the *NT5C2* homologue *CG32549* in *D. melanogaster* was associated with abnormal climbing behavior, more specifically, when driven by a neuronal promoter, supporting a role for *NT5C2* in motility. It is unrealistic to correlate fly motility with complex psychomotor disturbances experienced by human patients, but our results corroborate previous genetic associations between *NT5C2* and diseases associated with motor symptoms 1, 2, 3, 4, 5, 6. The effect of *CG32549* could be mediated by regulation of AMPK signaling and RPS6 activation, which are implicated in Drosophila motility 61, 62. A study showed that *CG32549* is downregulated in a Drosophila model of seizure (28), and work by another group demonstrated that distance moved during seizure-like activity can be reduced (rescued) upon AMPK activation using the drug metformin (29).

Limitations of our study should be acknowledged. First, we obtained a modest knockdown of *NT5C2* in hNPCs, which we hypothesize to be due to the proliferative nature of these cells. To support the link between *NT5C2*, AMPK signaling, and RPS6 activation, we overexpressed *NT5C2* in HEK293T cells and confirmed the differential regulation of AMPK and RPS6. Second, we observed a modest overlap of differentially expressed genes between siRNA treatments, which we hypothesize to be due to the low specificity associated with the siRNAs. This could be overcome by using a more effective gene silencing method, such as clustered regularly interspaced short palindromic repeats (CRISPR) interference (63). Third, we quantified the rate of protein translation using relative abundance of phosphorylated RPS6, but we did not investigate overall protein synthesis using methods such as the surface sensing of translation (SUnSET) (64). We have, however, provided support for the role of *NT5C2* in protein translation at the transcriptional level using our microarray and RT-qPCR data.

By exploring the role of *NT5C2* in neurobiology, we observe that the study of individual risk factors for complex disorders has the potential to advance our understanding of common biological pathways contributing to disease. Functional studies using model organisms or cell culture may not entirely capture the heterogeneity and complexity of psychiatric disorders but may provide insights to accelerate the identification of novel drug targets and biomarkers for psychiatric disorders.
