# Supplementary material for: The Psychiatric Risk Gene NT5C2 Regulates Adenosine Monophosphate-Activated Protein Kinase Signaling and Protein Translation in Human Neural Progenitor Cells
Source: Biol Psychiatry. 2019 Jul 15;86(2):120–30. doi: 10.1016/j.biopsych.2019.03.977 (PMC6614717; doi:10.1016/j.biopsych.2019.03.977)
Supplement: Supplemental Material [file mmc1.pdf]

The Psychiatric Risk Gene *NT5C2* Regulates Adenosine Monophosphate-Activated Protein Kinase Signaling and Protein Translation in Human Neural Progenitor Cells

Supplemental Information

Supplemental Methods and Materials

Brain samples

Human dorsolateral prefrontal cortex (DLPFC) sections (Brodmann Area 9) were obtained from the Medical Research Council London Neurodegenerative Diseases Brain Bank at the Institute of Psychiatry, Psychology & Neuroscience, King's College London. Mean age from subjects was 71.6 years and ranged from 18-92 years; this cohort consisted of 5 females and 2 males. All subjects were free from psychiatric or neurological diagnosis at the time of death.

Immunohistochemistry/fluorescence

To identify cell types associated with *NT5C2* expression, human brain sections were immunolabelled with an antibody raised against the NT5C2 protein. Paraffin-embedded sections (7 µm) were deparaffinized (2x 5 min xylene, 5 min IMS, 5 min 95% IMS, 70% IMS, water), and submitted to an antigen retrieval protocol (simmer cooking on microwave for 10 min in citrate buffer, pH 6). For immunohistochemistry using chromogenic labelling, the activity of endogenous peroxidases was blocked prior to chromogenic reaction. Sections were submitted to a perm/blocking step by incubation with normal rabbit serum 10% in phosphate buffer saline (PBS) containing 0.1% Triton-X for 2 hours. Next, sections were incubated with the primary antibody

(1:200) overnight at 4ºC in normal rabbit serum 10% PBS, followed on the next day by chromogenic detection using the Vectastain ABC Horseradish Peroxidase Kit (Vector Labs, Peterborough, United Kingdom), according to the manufacturer's protocol. Sections were counterstained for Nissl substance using 0.1% cresyl violet solution (w/v) at 60ºC for 10-15 min. Slides were mounted using DPX Mountant (Sigma-Aldrich, St. Louis, United States).

For immunofluorescence staining, sections were deparaffinized, submitted to antigen retrieval protocol, autofluorescence removal protocol (40 min incubation in aqueous solution of 0.25% potassium permanganate, followed by brief incubation with 1% oxalic acid), blocked using 5% normal goat serum (NGS) PBS containing 0.1% Triton-X, and incubated overnight at 4ºC with primary antibodies in blocking solution. On the next day, 3x PBS washes (10 min) were performed, followed by incubation with secondary antibodies for 2 hours at room temperature, followed with 3x 10 min PBS washes. Primary antibodies used included IBA1 (1:100 dilution, MP-290-CR01, Menarini Diagnostics, Winnersh, United Kingdom), GFAP (1:200 dilution, Z0334, Dako Agilent, Santa Clara, United States), MAP2 (1:500 dilution, ab5392, Abcam, Cambridge, United Kingdom), Parvalbumin (1:250 dilution, ab32895, Abcam), and NT5C2 (1:200 dilution, H00022978-M02, Abnova, Taipei, Taiwan), and secondary antibodies were Alexa 488, 568 and 633 antibodies (1:500 dilution, Thermo Fisher Scientific, Waltham, United States), in 5% NGS PBS. IF sections were mounted using Prolong Gold Antifade Reagent with Dapi (Thermo Fisher Scientific). Staining controls (no primary antibodies) did not show fluorescence upon imaging of sections using the imaging settings used per experiment. Quantification of co-localization of NT5C2 with MAP2, Parvalb, IBA1 and GFAP was performed using a custom ImageJ macro (1). Total NT5C2 intensities

(mean grey value) and NT5C2 intensities co-localized with neurons, interneurons, microglia, or astrocytes, were measured and calculated on z-projected image stacks using the immunolabelled-stained sections. Gaussian filter (0.5), background subtraction (50) and a threshold (Default) were applied to the z-projected images for each channel. The settings for NT5C2 were kept constant between the different co-localization experiments whilst the setting for MAP2, Parvalbumin, GFAP and IBA1 staining were adjusted for an optimal representation of neurons, interneurons, astrocytes and microglia, and then kept constant during image analyses. Co-localization was defined as pixel clusters in the NT5C2 channel that overlapped with pixel clusters in the MAP2, Parvalbumin, GFAP and IBA1 channels, with a set size cut-off at 0.05 μm^2. Both total NT5C2 and co-localized NT5C2 intensity were averaged over the 20 images taken per technical replicate (20 fields of view), per patient (n = 4 unaffected controls).

Cell lines

The conditionally-immortalized neural stem cell line **CTX0E16** was used to investigate global gene expression changes caused by the knockdown of the schizophrenia susceptibility gene *NT5C2*. This cell line was kindly provided by ReNeuron (www.reneuron.com) as part of a Material Transfer Agreement. This cell line was derived from the brain of a 12-week male fetus as detailed elsewhere (2, 3). Cells were routinely maintained at the proliferative stage, or terminally differentiated into neurons for 28 days (DD28), as described elsewhere (3, 4). Cultures were kept at 37^oC, with 5% CO_2, and split using Accutase when necessary. The proliferative state was maintained by culturing cells in DMEM:F12 (Sigma) supplemented with 0.03% human serum albumin (PAA), 100 $\mu g.ml^{-1}$ apo-transferrin (Scipac), 16.2 $\mu g.ml^{-1}$ putrescine

(Sigma), 5 µg.ml^{-1} human insulin (Sigma), 60 ng.ml^{-1} progesterone (Sigma), 2 mM L-glutamine (Sigma) and 40 ng.ml^{-1} sodium selenite (Sigma), 10 ng.ml^{-1} human FGF2, 20 ng.ml^{-1} human EGF (PeproTech) and 100 nM 4-hydroxy-tamoxifen (4-OHT) (Sigma). Terminal differentiation of CTX0E16 cells was achieved by replacing DMEM:F12 medium with Neurobasal Medium supplemented with B27 serum-free supplement (Life Technologies), 0.03% human serum albumin, 100 µg.ml^{-1} apo-transferrin, 16.2 µg.ml^{-1} putrescine, 5 µg.ml^{-1} human insulin, 60 ng.ml^{-1} progesterone, 2 mM L-glutamine, and 40 ng.ml^{-1} sodium selenite. Neural progenitor cells were grown laminin-coated (1 µg/cm^2) T75 Nunc flasks, or glass coverslips (No. 1.5, 13 mm) until fixed. Differentiated cultures were grown on coverslips coated with poly-D-lysine (0.2 mg.ml^{-1}, Sigma) and laminin.

Human induced pluripotent stem cells (**hiPSCs**) were generated from primary keratinocytes as previously described (5). Briefly, 1 x 10^5 primary hair root keratinocytes were transduced with Sendai virus expressing OCT4, SOX2, KLF4 and C-MYC (kind gift of M. Nakanishi, AIST Japan) (6, 7). Transduced keratinocytes were plated onto an irradiated MEF feeder layer (Millipore) in supplemented Epilife medium for ten days before switching to 'hES media' consisting of KO-DMEM/F12 supplemented with 20% Knock-out serum replacement, Non-essential amino acids, Glutamax, b-mercaptoethanol (all from Life Technologies) and bFGF (10 ng.ml^{-1}; Peprotech). After a further two weeks, reprogrammed colonies were picked straight into E8 media (Life Technologies) on Geltrex coated plasticware. HiPSCs were validated by genome-wide expression profiling using Illumina Beadchip v4 and the bioinformatics tool 'Pluritest' (8), embryoid body formation and tri-lineage differentiation potential, markers of pluripotency including Nanog, OCT4, SSEA4 and TRA1-81,

Alkaline phosphatase expression (Millipore), and for genomic stability by G-banded karyotyping (4). HiPSCs were maintained in E8 media on Geltrex-coated plasticware (Thermo Fisher). Neuronal differentiation of hiPSCs was achieved by replacing E8 medium on confluent (>95%) hiPSCs with neuralization medium: 1:1 mixture of N2- and B27-containing medium supplemented with 5 $\mu g.ml^{-1}$ insulin, 1 mM l-glutamine, 100 µm non-essential amino acids, and 100 µM 2-mercaptoethanol. At this stage, neuralization medium was further supplemented with 1 µm Dorsomorphin (Sigma), and 10 µm SB431542 (Cambridge Bioscience) to inhibit TGFβ signaling during neural induction (9, 10). Cells were maintained in this medium until the appearance of neuroepithelial cells (day 8), at which time cells were passaged using Accutase (Sigma) and maintained in neuralization medium. Neuroepithelial cells were grown and passaged 3 times, until neural rosettes (neural progenitor cells (NPCs); ~day 17) formed.

HEK293T cells were routinely maintained in T75 Nunc flasks (Thermo Fisher Scientific) in Advanced DMEM media supplemented with 10% fetal bovine serum and 2 mM L-glutamine (Thermo Fisher Scientific), and passaged every 2-3 days using Accutase when confluency was achieved. All cell lines were kept at 37°C, 5% CO_2.

In vitro transfections

<u>RNAi:</u> We used the Trilencer 27-mer *NT5C2* siRNA kit (SR307908, Origene, Rockville, MD, USA) to knockdown full length *NT5C2,* RefSeq transcripts NM_012229 and NM_001134373, using the double stranded RNA (dsRNA) sequences A (UGAGAAGUAUGUAGUCAAAGAUGGA) and B (ACAACUGUAAUAGCUAUUGGUCUTC), which target the most 5' exon of both

known *NT5C2* transcripts, according to the hg19. These dsRNAs were transfected in parallel with a negative scramble control sequence (SR30004, CGUUAAUCGCGUAUAAUACGCGUAT, sense sequence), and a fluorescently labelled short oligonucleotide (BLOCK-iT™ Fluorescent Oligo, Thermo Fisher Scientific). A million cells of CTX0E16 neural progenitors were plated in T75 flasks 24 hours before transfection (for RNA/protein extraction), or 10,000 cells in 13 mm coverslips (for immunocytochemistry assays). Four biological replicates (n=4) were transfected with 10 nM siRNAs in parallel with cultures transfected with a scramble siRNA (n=4) using N-TER Nanoparticle siRNA Transfection System (Sigma-Aldrich). Cells transfected with the positive siRNA control were manually counted in an Olympus IX70 fluorescence microscope using bright field visualization and a 568 nm filter to ascertain the level of transfection.

Overexpression: For overexpression experiments in HEK293T cells, transfection was performed using Lipofectamine 2000 (Thermo Fisher Scientific), according to the manufacturer's protocol. Briefly, $6.25x10^5$ cells were seeded into 6-well plates, transfected overnight with 6 µL Lipofectamine, 2.5 µg DNA in 500 µl OPTIMEM and 1.5 mL feeding media, and incubated in fresh medium for another day.

RNA and protein extraction

Total RNA and protein were extracted in parallel by detaching cells using Accutase, and splitting the obtained cell suspension into two tubes for harvesting. Tri-Reagent (Thermo Fisher Scientific) was used for total RNA extraction, according to the manufacturer's protocol. All samples showed absorbance ratios between 1.8 and 2.1 in a spectrophotometer ND-1000 (NanoDrop). For protein extraction, the pellet was

manually disrupted in RIPA Buffer (Thermo Fisher Scientific) containing 1x Halt Protease and Phosphatase Inhibitor Cocktail (Thermo Fisher Scientific), according to manufacturer's instructions, followed by three 30 sec 50% power sonication pulses intercalated with 30 sec rest in ice, incubation at 4ºC during 15 min, and centrifugation at 14,000 xg for 15 min to pellet out cell debris. Protein extracts were quantified using the Pierce™ BCA Protein Assay Kit (Thermo Fisher Scientific). RNA and protein samples were stored at -80ºC.

Quantitative reverse transcription polymerase chain reaction (RT-qPCR)

Total RNA samples were treated using the TURBO DNA-free kit™ (Thermo Fisher Scientific), according the manufacturer's protocol, using the DNase Inactivation Reagent. This RNA did not yield a PCR product in the absence of a reverse transcription step. Reverse transcription was performed using 1.5 µg of DNA-free RNA using SuperScript III® (SSIII) Reverse Transcriptase and RNaseOUT™ Recombinant Ribonuclease Inhibitor (Thermo Fisher Scientific), according to the manufacturer's instruction. The resulting cDNA was diluted 1:7 in RNAse-free water for the RT-qPCR reactions and stored at -20ºC. Quantitative PCR was performed in 12.5 µl reactions consisting of 5 µL diluted cDNA, HOT FIREPol EvaGreen qPCR Mix Plus (Solis BioDyne, Tartu, Estonia) and 200 nM primers using a real-time thermocycler MJ Research Chromo 4 (Bio-Rad, Hercules, CA, USA) linked to an MJ Opticon Monitor analytic software (Bio-Rad). The reaction program consisted of a 15 min incubation at 95ºC followed by 40 cycles of 20 sec at 95ºC, 20 sec at 60ºC, 20 sec at 72ºC, and by a melting curve analysis (temperature gradient from 60ºC to 95ºC). qPCR reactions were performed in duplicates or triplicates, with standard deviation between cycle threshold (C_t) for duplicates below 0.2. Melting curves revealed a single, clear peak for

each primer set. All primer sets had PCR efficiencies between 1.8 and 2.1, as determined by standard curves of four pooled cDNA dilution points. Prior to microarray analysis, a screening for the most stably expressed housekeeping genes (HKs) upon knockdown of *NT5C2* was performed using NormFinder (11). Primers are available on **Supplemental Table S3**. A screening for the most stably expressed HKs upon knockdown of NT5C2 was performed in cultures submitted to the siRNA treatments. The tested genes included *B2M* (beta-2-microglobulin), *RPL13A* (ribosomal protein L13a), *ACTB* (beta-actin), *SDHA* (succinate dehydrogenase complex, subunit A), *UBC* (ubiquitin C), *GAPDH* (glyceraldehyde-3-phosphate dehydrogenase) and *RPL30* (ribosomal protein L30), for which the most stably expressed genes were *RPL13A* and *B2M* (Supplemental Figure S8). Primers were designed using Primer3 (12) and purchased from Integrated DNA Technologies (London, UK). The mean C_t values for *B2M* and *RPL13A* were subtracted from the mean C_t value of each of the target genes, to generate normalized DC_t values (13). DDC_t values were then calculated by subtracting the average DC_t in a calibrator sample (scramble) from the average DC_t in each test sample (knockdown). The comparative $2^{-\Delta Ct}$ method (13) was used to generate relative gene expression data for use in the statistical analyses. Differential expression ($P < 0.05$) was assessed using a linear model, with normalized relative expression as the outcome variable, knockdown or scramble as the independent variable, co-varying for biological replicates and qPCR batch (different plates). Graphs were generated on GraphPad Prism 6 (GraphPad Software, La Jolla, CA, US).

Western blotting

Protein samples were diluted into 1x Laemmli sample buffer (Bio-Rad, Hercules, California, USA) containing beta-mercaptoethanol. Five µg of total protein were loaded

in 15-well 4–20% Mini-PROTEAN® TGX™ Precast Protein Gels (Bio-Rad), as described in Deans and collaborators (4). Nitrocellulose membranes were blocked in milk 5% TBS-T (20 mM Tris, 150 mM NaCl containing 0.1% Tween-20), and incubated overnight at 4ºC with primary antibodies in blocking solution. Primary antibodies used included the AMPK-alpha (AMPK-alpha D6 antibody sc-74461, dilution 1:200 in milk blocking buffer, Santa Cruz Biotechnology, Dallas, TX, USA), phospho-AMPK-alpha (p-AMPK alpha1/2 Thr 172, sc-33524, dilution 1:200 in milk blocking buffer, Santa Cruz Biotechnology), the NT5C2 monoclonal antibody (M02, clone 3C1, dilution 1:200 in milk blocking buffer, Abnova Corporation, Taipei, Taiwan), total rpS6 (clone 54d2, dilution 1:1000 in milk, Cell Signalling, Danvers, Massachusetts, United States) and phospho-rpS6 (p-rpS6 Ser 235/236, 1:200, Cell Signalling). Secondary antibodies used were the Alexa Fluor 680 goat-anti-mouse, and Alexa Fluor 790 goat-anti-rabbit at 1:10,000 dilution (Thermo Fisher Scientific). Membranes were scanned in an Odyssey (LI-COR) and regions of interest were drawn and quantified using the LI-COR® Odyssey® Software (LI-COR). Integrated intensity values for phosphorylated AMPK-alpha were normalized to total kinase amount, and again to the scramble treated condition; total AMPK was normalized to B-actin, and normalized to the scramble condition.

Immunocytochemistry

We used immunocytochemistry to validate the knockdown in hNPCs and visualize NT5C2 sub-cellular distribution in these cells. Neural progenitor cells (CTX0E16) were seeded in 13 mm coverslips (10.000 cells/well) in four biological replicates (n=4). Cultures were transfected with 10 nM siRNAs A and B in parallel with cultures transfected with a scramble siRNA (n=4) using N-TER Nanoparticle siRNA

Transfection System (Sigma-Aldrich). Coverslips were immunostained for NT5C2 using the mouse monoclonal NT5C2 antibody (M02, clone 3C1, Abnova) to quantify NT5C2 intensity, and beta-3-tubulin (1:500, polyclonal, Abcam ab41489), to define the cellular space. Cells were permeabilized and blocked in 2% normal goat serum (NGS) PBS-T for one hour at room temperature. Primary antibodies were incubated overnight at 4ºC in blocking solution at their respective dilutions. Cells were washed off three times with PBS-T on the next day, and incubated with secondary antibodies diluted 1:1,000 for one hour at room temperature (Alexa Fluor goat fluorescent secondary antibodies specific to mouse, rabbit or chicken, Thermo Fisher Scientific). Cells were washed in PBS-T three times, stained for DAPI and mounted using ProLong Gold Antifade Mountant (Thermo Fisher Scientific). Fluorescently stained cells were imaged in a Leica SP5 confocal microscope using the 40x objective, at the King's College London Wohl Cellular Imaging Centre, London, United Kingdom. Laser power, pinhole and smart gain were adjusted using control cultures during the first rounds of imaging acquisition, and were kept constant across individual experiments. Staining controls (no primary antibodies) did not show fluorescence using the confocal power and pinhole settings used in each experiment. Images were taken as z-stacks containing 10 plans (z-step = 0.5 μm), and exported to ImageJ (14), where background subtracted images and maximum intensity projections were generated (15). Cell morphology was determined by thresholding the beta-3-tubulin channel, so that individual cell somas were delimited as regions of interest (ROI). All images were converted to grey scale, and corrected total cell fluorescence (CTCF) for NT5C2 was calculated as: CTCF = integrated density - (area x mean fluorescence of three background readings). Each culture was imaged in four fields of view (FOV), and CTCF values were obtained for 20 cells per FOV, in four biological replicates (320 cells/condition).

Microarray analysis

Samples were submitted for microarray analysis at the Institute of Psychiatry, Psychology and Neuroscience (IoPPN) Genomics & Biomarker Core Facility, located at the Social, Genetic & Developmental Psychiatry Centre at King's College London, United Kingdom. RNA integrity was checked for all samples using the Agilent RNA 6000 Nano Kit on the Agilent 2100 Bioanalyzer (Agilent, Santa Clara, CA, USA). Microarray libraries were constructed using the Illumina® TotalPrepTM-96 RNA Amplification Kit (Illumina), in which biotin was used to label cRNA. Library was hybridized using the Whole-Genome Gene Expression Direct Hybridization (Illumina) Kit. The Illumina Human HT12 v4 BeadChip array (Illumina, Cambridge, Cambridgeshire, UK) was scanned on the Illumina HiScan. Microarray probe raw fluorescence intensities were obtained using the GenomeStudio Software (Illumina). Background correction and log2 transformation were applied to the data using the lumi Bioconductor package (16) in RStudio. Probe intensities were analyzed in a linear regression model to measure the effect of the siRNAs while correcting for variability between biological replicates and microarray batches. To limit spurious results, probes that were not detected in all samples with a detection threshold $P < 0.05$ were discarded from the analysis. Differentially expressed genes (nominal significance, $P < 0.05$) showing the same direction of effect for both siRNA conditions were submitted to further analysis. The statistical significance for the overlapping gene list was calculated in R using the "GeneOverlap" package, which uses the Fisher's exact test to estimate the overlapping p-value considering all genes in the genome as background. The expression data were deposited in the GEO database under accession code GSE109240.

Gene ontology analysis

Gene ontology (GO) was performed to determine the biological processes associated with the knockdown of *NT5C2* in the neural stem cells. The overlapping gene set was subdivided into up- and downregulated gene sets, which were input into GeneMania 3.4.1 running on Cytoscape 3.4 (17), to compute networks of co-expression and co-localization, and to reveal enrichment of GO terms. The default background list for this analysis was used, which corresponds to all genes in the genome. Significant hits were defined as those that survived correction for multiple comparisons using the false discovery rate (FDR) at $q < 0.05$ (5%).

Fly stocks and CG32549 knockdown

To test the role of *NT5C2* in psychomotor behavior in *D. melanogaster*, the fly homologue of *NT5C2*, *CG32549*, was knocked down by crossing the CG32549-RNAi line (v30079) with upstream activating sequences (UAS) that promote activating Gal4 expression ubiquitously (*ACT5C:* BL4414), in neurons or neural progenitor cells (*ELAV:* BL8765), or in gut (*GUT:* DGRC113094). To account for the effect of temperature across conditions, and because the Gal4-UAS system is most active at 29°C (18), all crosses were incubated at this temperature from the pupal stage so that we obtained the strongest knockdown of *CG32549* under different RNAi expression at this specific point in development, when new neurons are still being formed (19). This allowed us to disentangle the potential function of *CG32549* in non-neuronal cells, such as muscles, which could influence performance in the negative geotaxis assay. The negative geotaxis assay was used to calculate climbing success, defined as the percentage of the initial 20 flies allocated per Falcon tube which climbed over an

arbitrary mark, to assess psychomotor behavior (n = 4+ flasks) (20). Survivorship was defined as the percentage of the initial 20 flies allocated per tube which were alive 17-20 days post eclosure (n = 4+). All statistic comparisons were made within UAS lines (e.g. UAS vs UAS-RNAi crosses), as the ratio of climbing success or flies alive in a UAS-RNAi line relative to the same UAS line without the RNAi cassette. All fly strains were maintained and amplified for experimentation with approximately 2.5 g of Carolina Formula 4-24 Instant *Drosophila* media (Carolina Biological Supply, North Carolina, United States), 10 mL of deionized water, and a dash of dry baker's yeast granules. Flies were routinely maintained at 25°C at a 12hr:12hr light-dark photoperiod.

***CG32549* knockdown validation**

Approximately 60 *D. melanogaster* fly heads were dissected per condition (ubiquitous knockdown vs. wild-type control). RNA extractions were carried out using PrepEase RNA Spin Kits by USB or Trizol Reagent, and eluted using molecular grade water. cDNA synthesis was carried using 300 ng of RNA with Applied Biosystems High Capacity Reverse Transcription cDNA Synthesis Kit following the manufacturer's protocol. cDNA samples were then diluted 1:10 times before proceeding to quantitative RT-PCR (RT-qPCR) analysis. All gene expression studies were carried out using Bio-Rad CFX96 Real-Time System. cDNA (1 µl) was incubated with NT5C2 specific primers and Bio-Rad SYBR Green Supermix. Results were measured using a threshold of 1000 relative fluorescence units (RFUs). Ct values were analyzed using the DC_t method, using RpL32 (CG7939) as reference (HK) gene. *CG32549* expression was represented as a % of expression in w^{1118} flies crossed with Act5c Gal4 driver, and analyzed using the unpaired t-test.

Negative geotaxis/climbing assay and survival analysis of D. melanogaster

Flies which had NT5C2 knocked down ubiquitously, in neurons, or in gut, were tested for differences in psychomotor behavior and survival. The negative geotaxis/climbing assay was based on Gargano and colleagues (20). Twenty male flies between 1-3 days post emergence days were place in a 50 mL Falcon tube and were allotted 30 minutes to acclimate to the environment. Flies were forcefully pushed to the bottom of the Falcon tube, and the number of flies that had climbed above the 30 mL line after six seconds was quantified and represented as a percentage of the total flies in the tube. Each CG32549-RNAi line crossed with its tissue specific Gal-4 driver was normalized to the same line without the RNAi cassette. Survivorship was determined at 17-20 days post emergence, as the number of flies alive out of the 20 flies per tube.

Supplemental Figures

NT5C2 expression (mouse cortex single cell RNA-sequencing)

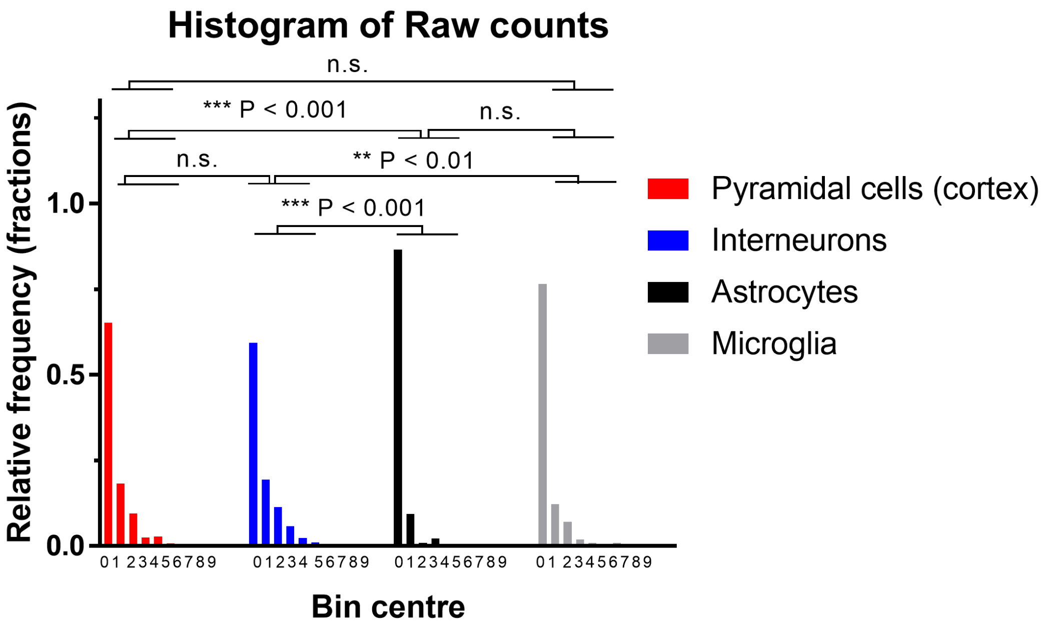

Supplemental Figure S1. Relative frequency of raw read counts from the single-cell RNA-sequencing data obtained from the mouse brain. This analysis suggests that *NT5C2* expression occurs in a cell-type specific manner (Kruskal-Wallis test, $P < 0.001$). Post-hoc analysis confirmed that *NT5C2* was more abundant in pyramidal neurons than in astrocytes (Dunn's corrected $P < 0.001$), and more abundance in interneurons than microglia (corrected $P < 0.01$) or astrocytes (corrected $P < 0.001$). Source: Amit and colleagues (21).

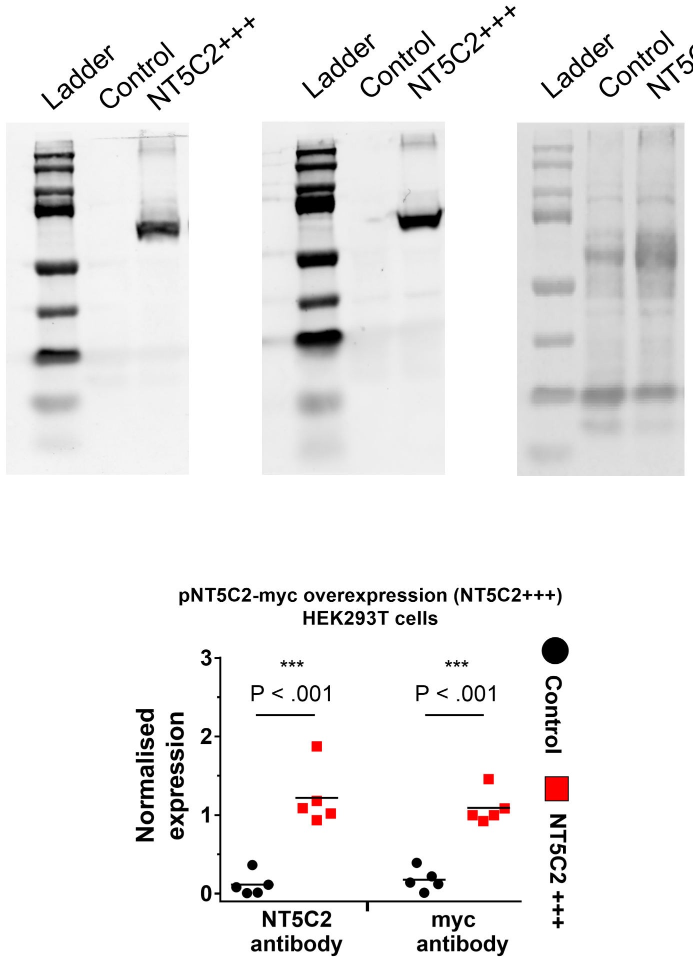

Supplemental Figure S2. NT5C2 antibody validation by overexpressing pNT5C2-myc in HEK293T cells. The overexpression was detected using NT5C2 and myc antibodies. There was a significant effect of transfection on immunodetection of NT5C2 and myc (independent t-tests; *NT5C2:* t(8) = 6.10, P < .001, Bonferroni corrected P < .001; *myc:* t(8) = 8.02, P < .001, corrected P < .001).

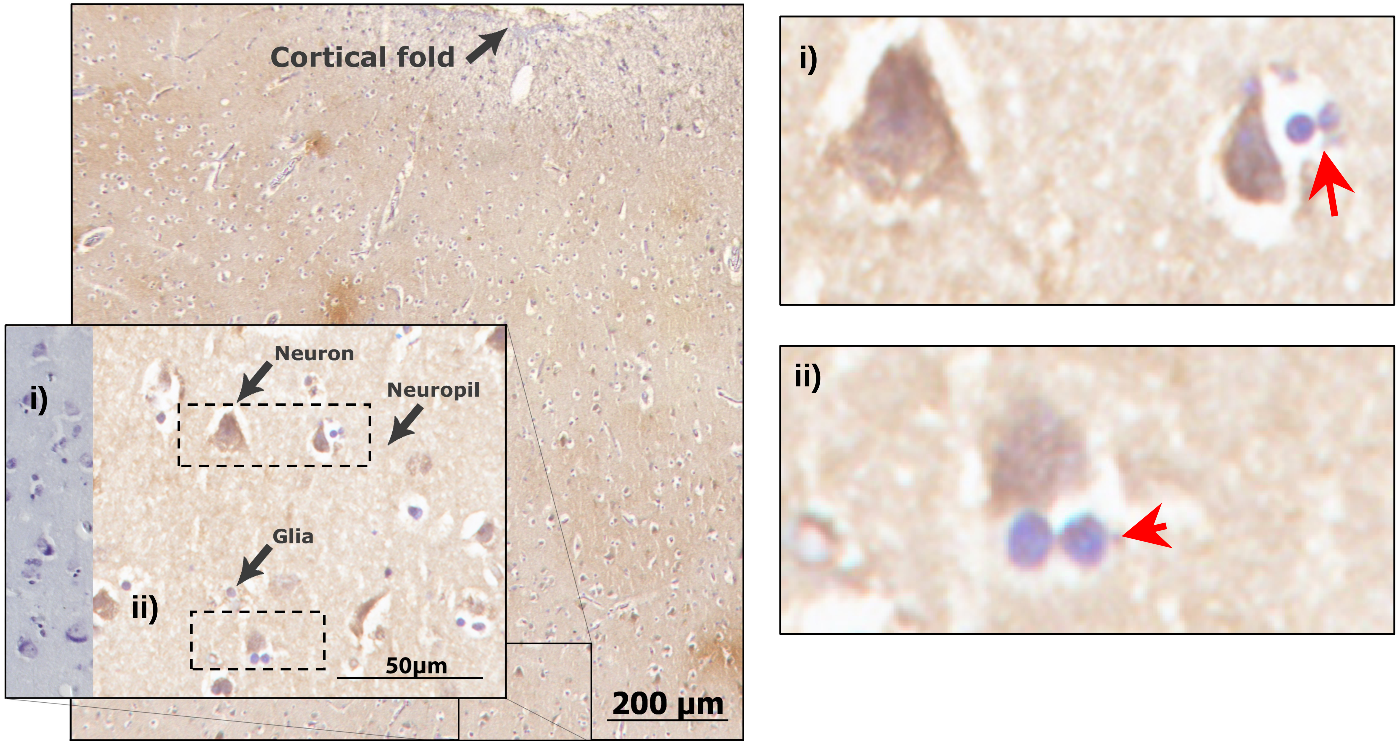

Supplemental Figure S3. Distribution of the NT5C2 protein in the adult brain. **(A)** Immunolabelled tissue counterstained with Nissl, demonstrating presence of NT5C2 in neurons, glial cells and neuropil (black arrows), and absence in glial cells (red arrows). Negative immunostaining control (no primary antibody) is shown on the left.

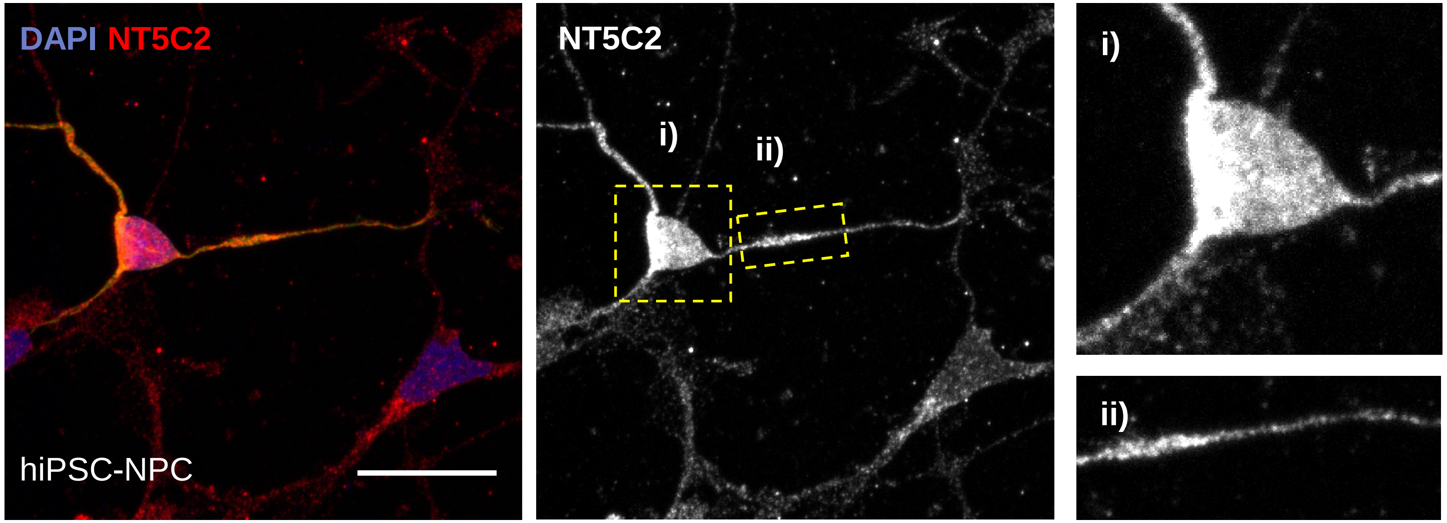

Supplemental Figure S4. Validation NT5C2 antibody. CTX0E16 hNPCs were transfected with the pNT5C2-myc plasmid and immunostained with an antibody for NT5C2. Both exogenous and endogenous NT5C2 could be detected; staining for exogenous NT5C2 was significantly higher compared to staining for endogenous protein. Yellow dashed boxes show high magnification zoom ins demonstrating the exogenous NT5C2 localizes within cell soma and along processes. Scale bar = 20 µm.

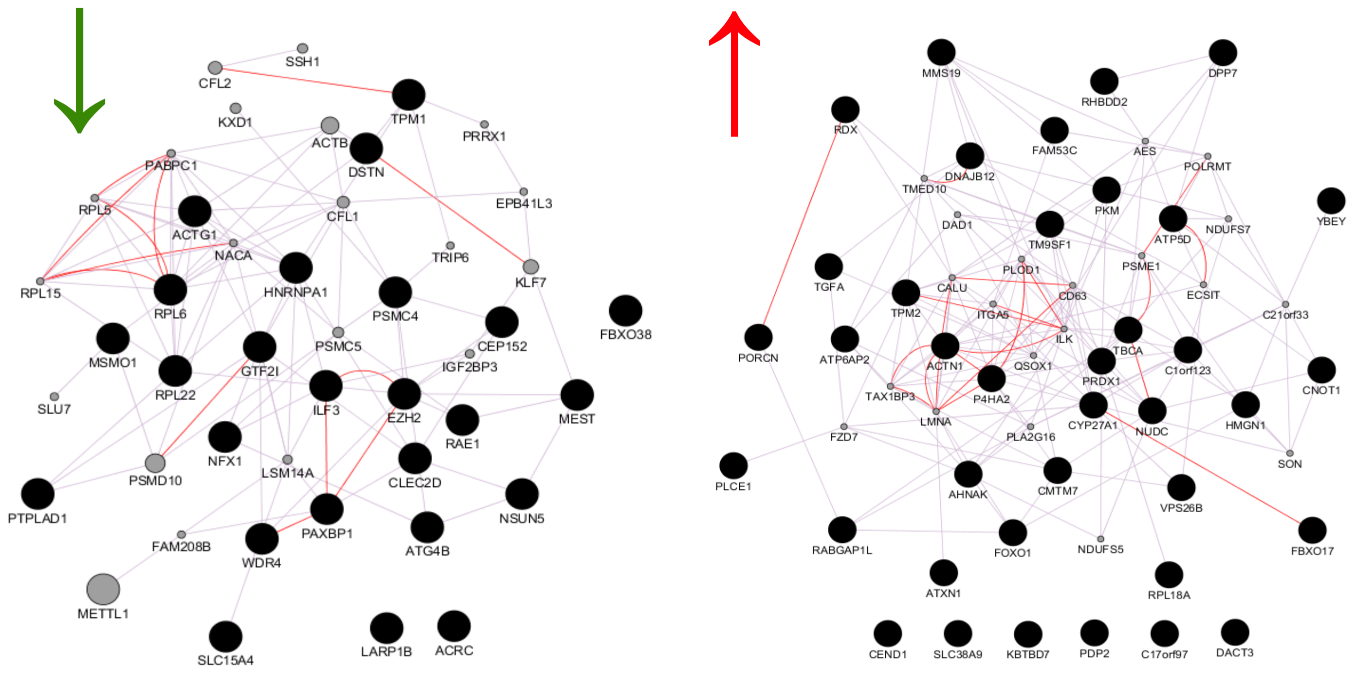

Supplemental Figure S5. Gene expression networks associated with the knockdown (left: downregulated; right: upregulated). Connecting lines define co-expression (purple) and co-localization (red). Images were generated with GeneMania (22).

A.

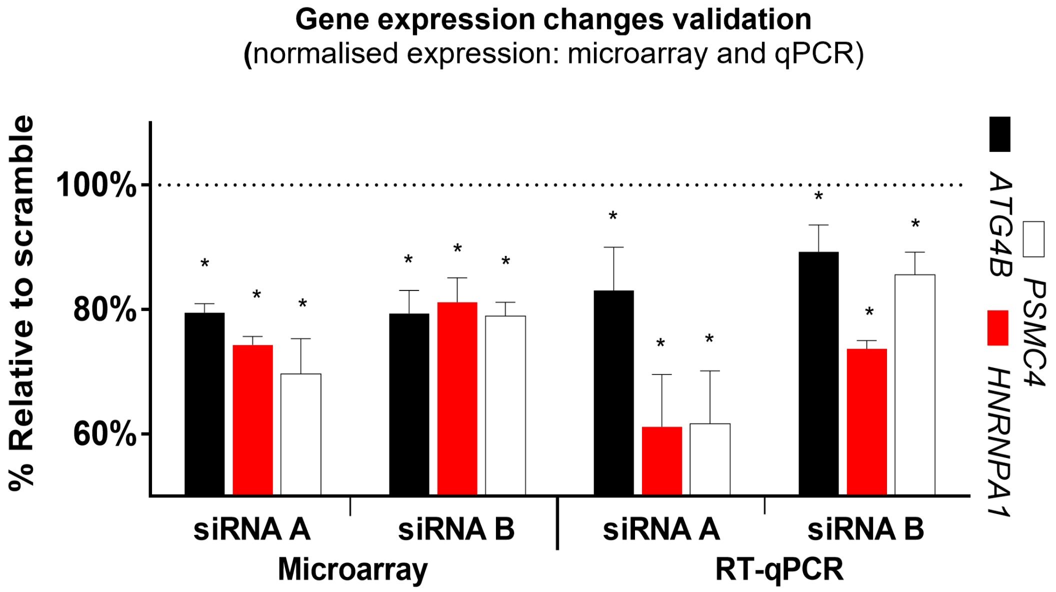

B.

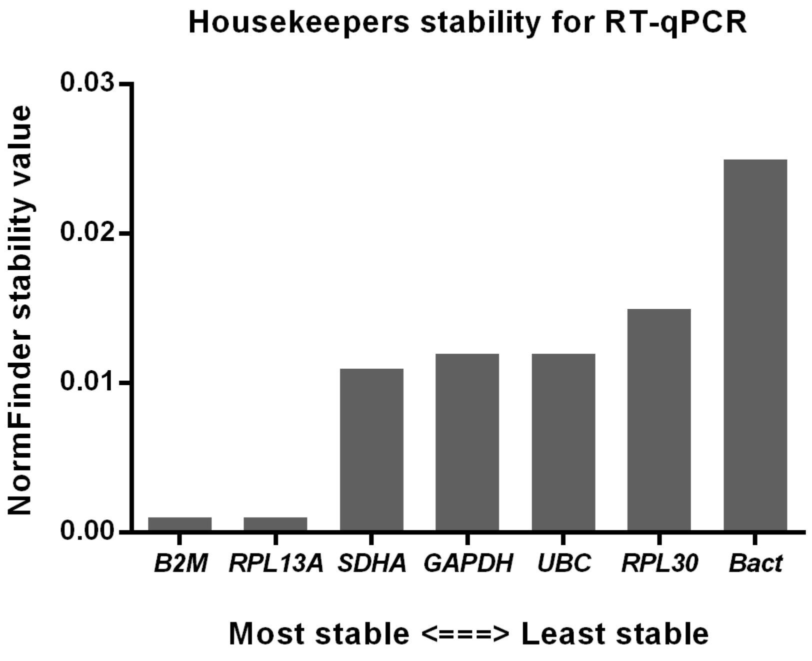

Supplemental Figure S6. RT-qPCR validation of gene expression changes captured in the microarray analysis. **(A)** There was a significant effect of the knockdown on the downregulation of a panel of genes related to the GO terms implicated in the knockdown: the heterogeneous nuclear ribonucleoprotein A1 gene (*HNRNPA1*; siRNA A: 61.19 ± 14.53, t(5) = 5.53, P = .003, Bonferroni corrected P = .018; siRNA B: 73.71

± 2.67, t(6) = 19.70, P < .001, corrected P < .001), which is implicated in protein translation and amyotrophic lateral sclerosis (23); the proteasome 26S subunit, ATPase 4 gene (*PSMC4;* siRNA A: 61.64 ± 14.71, t(5) = 5.40, P = .003, corrected P = .018; siRNA B: 85.62 ± 7.18, t(6) = 4.01, P = .007, corrected P = .042), which is implicated in protein degradation and cytoskeleton remodeling (24), and in Parkinson's disease (25), and the autophagy-related cysteine peptidase gene (*ATG4B;* nominal significance only; siRNA A: 83.06 ± 12.09, t(5) = 2.90, P = .034; siRNA B: 89.26 ± 8.69, t(6) = 2.47, P = .048), which is implicated in the regulation of the cytoskeleton (26). **(B)** Stability of expression of potential housekeeping (HK)/reference genes upon knockdown of *NT5C2* in hNPCs, according to stability value scored by NormFinder. CT values for these genes were obtained in knockdown and control cultures, and analyzed with this tool. *B2M* and *RPL13A* were chosen as reference genes for RT-qPCR assays due to low variation in expression in control and knockdown cultures.

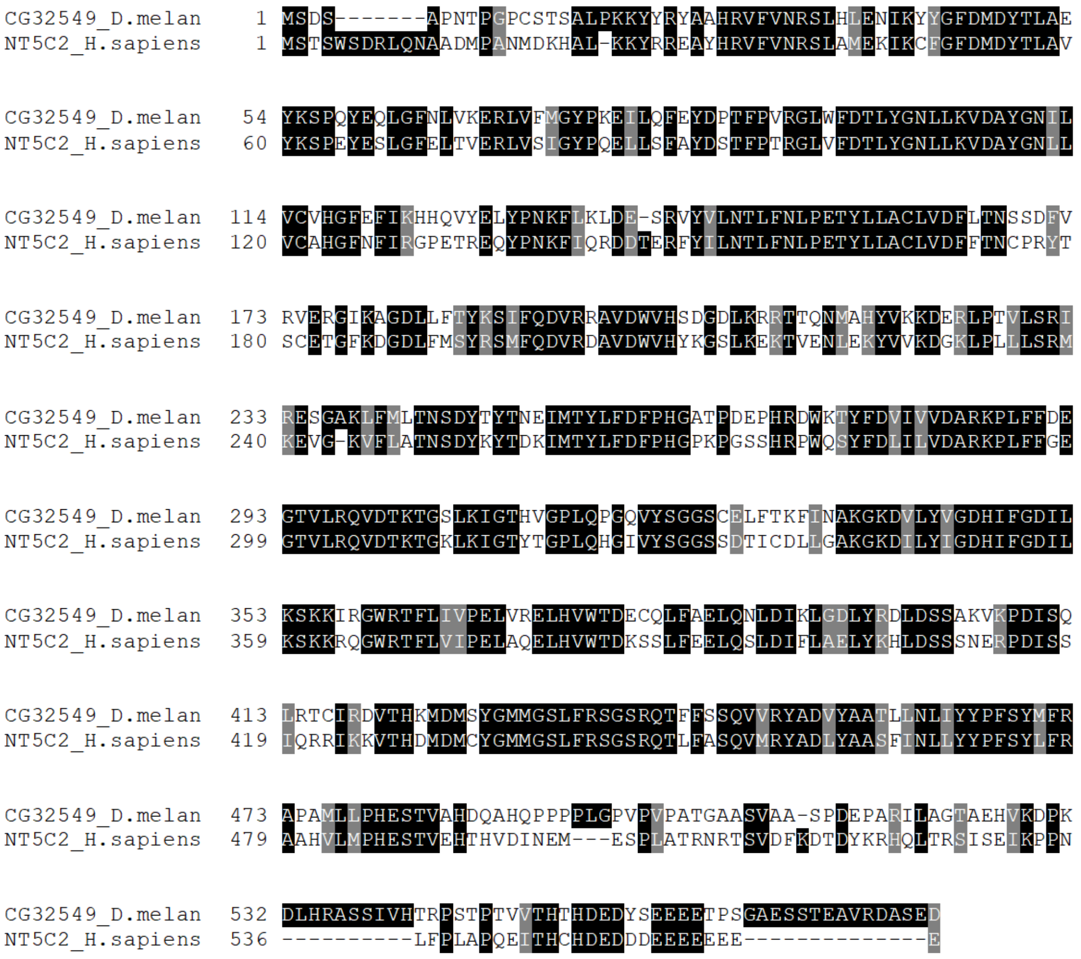

Supplemental Figure S7. Protein sequence alignment between NT5C2 (*Homo sapiens*) and CG32549 (*Drosophila melanogaster*), demonstrating high degree of homology between these proteins. They have been estimated to share approximately 60% identity and 80% similarity.

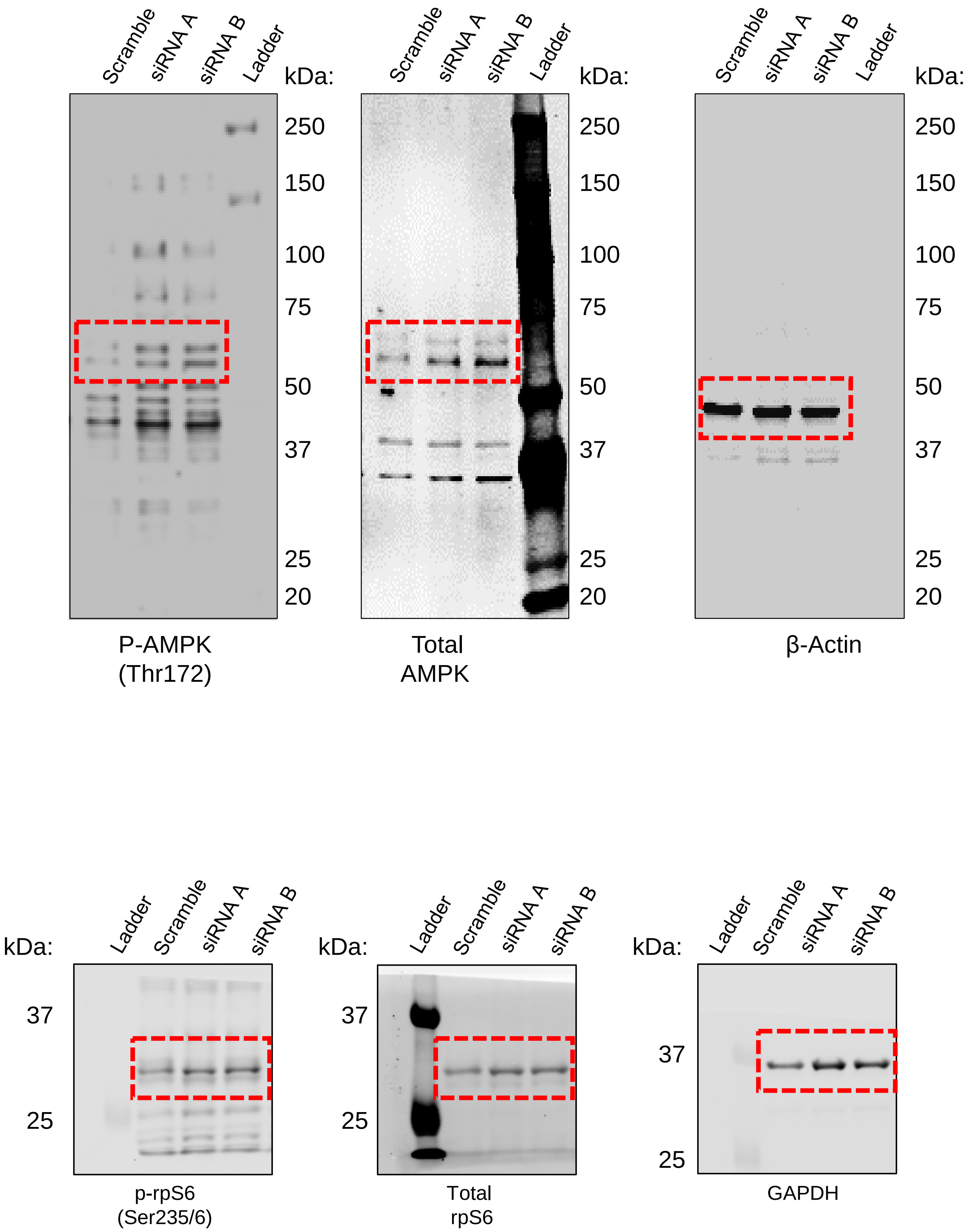

Supplemental Figure S8. Full length blots shown in Figures 3E and F.

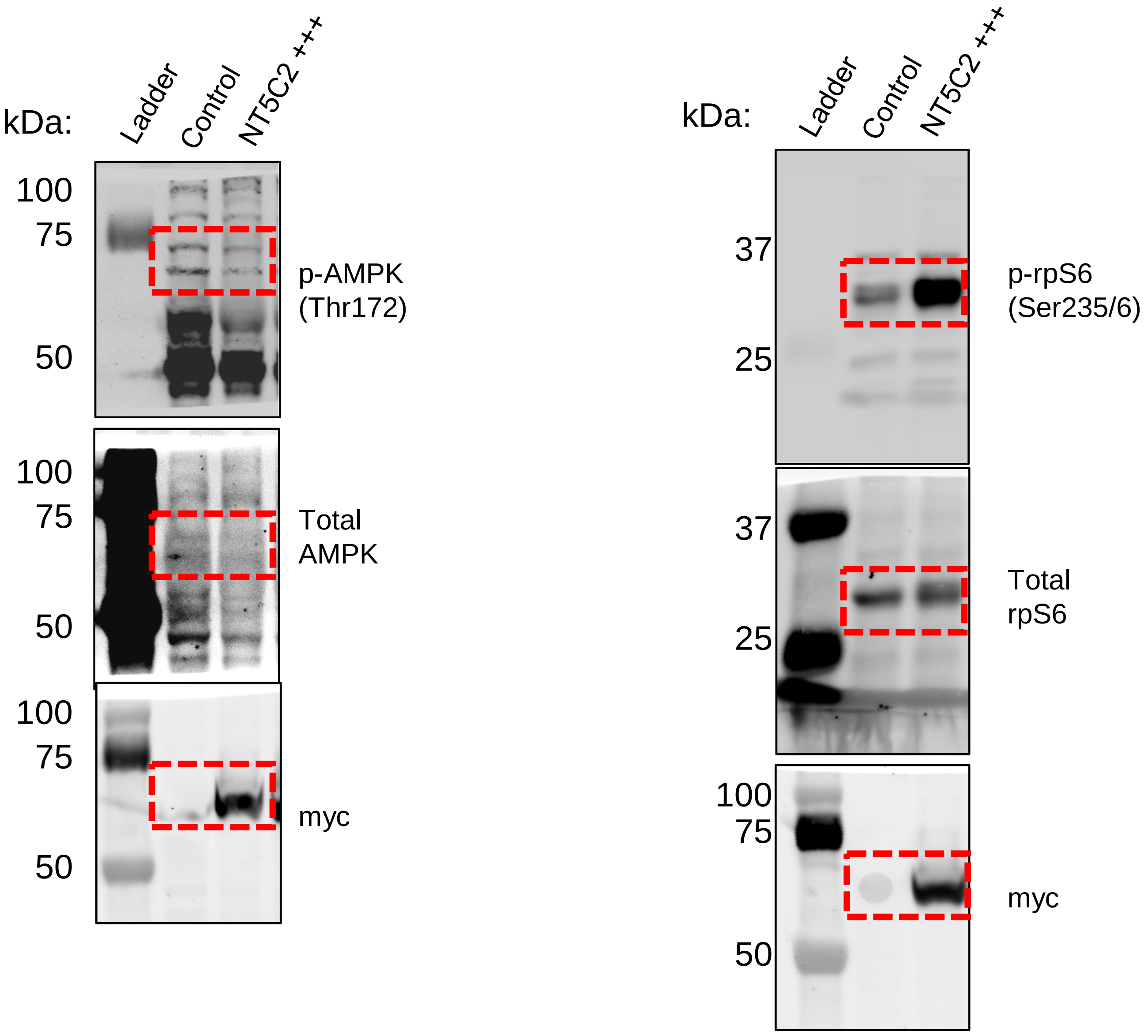

Supplemental Figure S9. Full length blots shown in Figure 3G.

Supplemental Table S1. Expression changes associated with the knockdown of NT5C2 in CTX0E16 hNPCs.

Gene symbol	Group A			Group B			Average uncorrect. P-val*
	Probe ID	P-val (uncorrect.)	Fold-change	Probe ID	P-val (uncorrect.)	Fold-change	
NUDC	6110477	0.003	1.106	6110477	0.007	1.168	0.005
CYP27A1	7040735	0.002	1.071	7040735	0.015	1.035	0.009
LOC729799	1400671	0.005	0.977	1400671	0.014	0.936	0.009
SC4MOL	4200037	0.014	0.849	4200037	0.006	0.83	0.01
ATG4B	2060136	0.006	0.795	2060136	0.014	0.791	0.01
TPM1	2140653	0.004	0.833	1010482	0.019	0.867	0.012
HNRNPA1	10209	0.004	0.743	10209	0.021	0.809	0.012
KBTBD7	7210280	0.007	1.232	7210280	0.018	1.059	0.013
PDP2	3390368	0.024	1.116	940600	0.001	1.074	0.013
CEND1	5560162	0.008	1.063	5560162	0.018	1.096	0.013
HMGN1	6220300	0.02	1.259	6220300	0.006	1.167	0.013
RPL6	2630561	0.002	0.858	1980520	0.024	0.975	0.013
TBCA	5960093	0.004	1.323	5960093	0.026	1.21	0.015
P4HA2	270408	0.025	1.08	270408	0.007	1.078	0.016
NFX1	4060494	0.021	0.867	4060494	0.012	0.884	0.016
PLCE1	6220687	0.028	1.151	6220687	0.005	1.082	0.016
ILF3	1450075	0.006	0.9	3360689	0.027	0.908	0.016
PRDX1	6180446	0.005	1.298	6180446	0.03	1.194	0.018
NSUN5	4730132	0.019	0.913	4730132	0.016	0.953	0.018
TPM2	1230451	0.032	1.039	1230451	0.006	1.062	0.019
RHBDD2	6650746	0.015	1.229	6650746	0.023	1.115	0.019
C21ORF57	6380187	0.026	1.075	2900403	0.013	1.078	0.019
HS.363526	3870025	0.034	1.141	3870025	0.005	1.068	0.019
LARP1B	5910164	0.014	0.83	150475	0.025	0.95	0.02
DPP7	1240047	0.001	1.056	670608	0.039	1.048	0.02
SLC38A9	7000184	0.038	1.064	7000184	0.002	1.062	0.02
ATP6AP2	7560097	0.006	1.284	7560097	0.035	1.282	0.021
PKM2	160170	0.008	1.197	160170	0.033	1.194	0.021
DSTN	4590470	0.007	0.944	4590470	0.035	0.949	0.021
RABGAP1L	360221	0.012	1.019	360221	0.031	1.017	0.022
TM9SF1	780296	0.021	1.139	780296	0.024	1.035	0.023
C20ORF199	2480064	0.033	1.197	2480064	0.013	1.136	0.023
PTPLAD1	6040048	0.003	0.967	6040048	0.043	0.934	0.023
PSMC4	150544	0.043	0.692	6180440	0.003	0.789	0.023
RPL18A	940332	0.047	1.062	3310487	<0.001	1.074	0.023
LOC150568	4220497	0.021	1.086	4220497	0.026	1.073	0.024
EZH2	60639	0.035	0.916	4180524	0.013	0.941	0.024
FAM53C	2650019	0.046	1.235	2650019	0.001	1.193	0.024
MEST	990070	0.014	0.905	990070	0.034	0.922	0.024
DNAJB12	5560484	0.037	1.051	5560484	0.012	1.046	0.024
DACT3	1990196	0.005	1.097	1990196	0.045	1.073	0.025
ATP5D	1110377	0.041	1.176	1110377	0.008	1.125	0.025
PORCN	3290343	0.018	1.039	3290343	0.032	1.059	0.025
CLEC2D	4590717	0.024	0.929	5490341	0.026	0.771	0.025
FBXO38	7570139	0.025	0.945	7570139	0.026	0.908	0.025

Gene symbol	Group A			Group B			Average uncorrect. P-val*
	Probe ID	P-val (uncorrect.)	Fold-change	Probe ID	P-val (uncorrect.)	Fold-change	
FOXO1	6770719	0.026	1.168	6770719	0.026	1.122	0.026
CMTM7	2140239	0.016	1.181	2140239	0.035	1.091	0.026
C17ORF97	4640161	0.038	1.09	4640161	0.014	1.111	0.026
AHNAK	450553	0.011	1.343	2810482	0.043	1.063	0.027
ACTN1	5080364	0.013	1.142	5080364	0.041	1.175	0.027
ATXN1	520601	0.011	1.153	520601	0.044	1.116	0.027
LOC440589	5870328	0.022	0.817	5870328	0.034	0.893	0.028
TGFA	3420296	0.042	1.215	3420296	0.016	1.154	0.029
ACRC	3850192	0.032	0.935	3850192	0.027	0.936	0.03
C21ORF66	6770753	0.016	0.951	6280626	0.045	0.859	0.031
CNOT1	6520382	0.031	1.091	6520382	0.032	1.187	0.032
RPL22	7380689	0.048	0.831	7380689	0.018	0.953	0.033
VPS26B	7100577	0.042	1.215	7100577	0.027	1.152	0.034
RDX	6110291	0.028	1.299	6110291	0.042	1.233	0.035
MMS19L	2230528	0.031	1.24	2230528	0.04	1.18	0.035
RAE1	4780541	0.049	0.948	4780541	0.027	0.936	0.038
GTF2I	4570736	0.027	0.805	4570736	0.049	0.861	0.038
WDR4	3930703	0.049	0.967	3930703	0.029	0.819	0.039
SLC15A4	160279	0.032	0.825	6650465	0.047	0.819	0.04
CEP152	4390176	0.047	0.901	990189	0.034	0.92	0.04
ACTG1	6510072	0.049	0.785	6510072	0.031	0.774	0.04
LOC646345	4730504	0.037	1.253	4730504	0.046	1.132	0.042
C1ORF123	5720414	0.041	1.059	5720414	0.043	1.124	0.042
FBXO17	2140047	0.044	1.065	2140047	0.048	1.032	0.046

Supplemental Table S2. Gene ontology (GO) terms associated with the knockdown of NT5C2 in neural stem cells.

Effect	GO terms	FDR	Genes
↓	GO:0005200 Structural constituent of cytoskeleton (O=4, C=67)	**0.047**	*ACTB, ACTG1, EPB41L3, TPM1*
↓	GO:0043624 Cellular protein complex disassembly (O=5, C=135)	**0.047**	*CFL2, RPL15, RPL22, RPL5, RPL6*
↓	GO:0000184 Nonsense-mediated mRNA decay (O=5, C=116)	**0.047**	*PABPC1, RPL15, RPL22, RPL5, RPL6*
↓	GO:0015934 Large ribosomal subunit (O=4, C=67)	**0.047**	*RPL15, RPL22, RPL5, RPL6*
↑	GO:0005924 Cell-substrate adherens junction (O=4, C=84)	0.593*	*ILK, ITGA5, RDX, ACTN1*

* Top upregulated non-redundant GO term (not significant after FDR correction).

Legend: number of reference genes in the category (C), number of genes in the gene set and also in category (O).

Supplemental Table S3. Oligonucleotide sequences used in the RT-qPCR assays.

Target	Forward 5'-3'	Reverse 5'-3'	Usage
NT5C2	CTCCCAACCTCTTCCCACTG	GGACCTCGTTTGTTCCTGTG	Overall NT5C2 expression
NM_001134373	CATATCTGCTGCATTCTGTAACCGA	GTGCGCTGGAGCCGAATAC	NT5C2 transcript expression
NM_012229	CCGAGGCGAATGGATCACTTG	GCGCTGGAGCCGAGTTTC	NT5C2 transcript expression
CG32549 (NT5C2)	GTCGCGTGTCTATGTCCTGAA	TCCTGGAAAATCGACTTGTAGGT	CG32549 expression *(*Drosophila*)*
CG7939 (RPL32)	GATGACCATCCGCCCAGCA	CGGACCGACAGCTGCTTGGC	Housekeeper (Drosophila*)*
ATG4B	TACTCTGACCTACGACACTCTCC	TGCTGTATTTTCTACCCAGTATCCA	Microarray analysis validation
PSMC4	GTCCTATCCTGCCCTTCCAG	GTGTGGCCTGGGATGATCT	Microarray analysis validation
HNRNPA1	CGGAGTCTACCAATGCCGAA	CAGAAAGGAGCAAGCTGACG	Microarray analysis validation
B2M	TATCCAGCGTACTCCAAAGA	GACAAGTCTGAATGCTCCAC	Housekeeper screening (tested/chosen)
RPL13A	CCTGGAGGAGAAGAGGAAAGAGA	TTGAGGACCTCTGTGTATTTGTC	Housekeeper screening (tested/chosen)
ACTB	TGTGATGGTGGGAATGGGTCAG	TTTGATGTCACGCACGATTTCC	Housekeeper screening (tested)
SDHA	TGGGAACAAGAGGGCATCTG	CCACCACTGCATCAAATTCATG	Housekeeper screening (tested)
UBC	ATTTGGGTCGCGGTTCTTG	TGCCTTGACATTCTCGATGGT	Housekeeper screening (tested)
GAPDH	GAAATCCCATCACCATCTTCCAGG	GAGCCCCAGCCTTCTCCATG	Housekeeper screening (tested)
RPL30	ACAGCATGCGGAAAATACTAC	AAAGGAAAATTTTGCAGGTTT	Housekeeper screening (tested)

Supplemental References

1. Notter T, Panzanelli P, Pfister S, Mircsof D, Fritschy JM (2014): A protocol for concurrent high-quality immunohistochemical and biochemical analyses in adult mouse central nervous system. *The European journal of neuroscience*. 39:165-175.

2. Pollock K, Stroemer P, Patel S, Stevanato L, Hope A, Miljan E, et al. (2006): A conditionally immortal clonal stem cell line from human cortical neuroepithelium for the treatment of ischemic stroke. *Exp Neurol*. 199:143-155.

3. Anderson GW, Deans PJ, Taylor RD, Raval P, Chen D, Lowder H, et al. (2015): Characterisation of neurons derived from a cortical human neural stem cell line CTX0E16. *Stem Cell Res Ther*. 6:149.

4. Deans PJM, Raval P, Sellers KJ, Gatford NJF, Halai S, Duarte RRR, et al. (2016): Psychosis risk candidate ZNF804A localizes to synapses and regulates neurite formation and dendritic spine structure. *Biological Psychiatry*.

5. Cocks G, Curran S, Gami P, Uwanogho D, Jeffries AR, Kathuria A, et al. (2014): The utility of patient specific induced pluripotent stem cells for the modelling of Autistic Spectrum Disorders. *Psychopharmacology (Berl)*. 231:1079-1088.

6. Nishimura K, Sano M, Ohtaka M, Furuta B, Umemura Y, Nakajima Y, et al. (2011): Development of defective and persistent Sendai virus vector: a unique gene delivery/expression system ideal for cell reprogramming. *J Biol Chem*. 286:4760-4771.

7. Takahashi K, Tanabe K, Ohnuki M, Narita M, Ichisaka T, Tomoda K, et al. (2007): Induction of pluripotent stem cells from adult human fibroblasts by defined factors. *Cell*. 131:861-872.

8. Muller FJ, Schuldt BM, Williams R, Mason D, Altun G, Papapetrou EP, et al. (2011): A bioinformatic assay for pluripotency in human cells. *Nat Methods*. 8:315-317.

9. Chambers SM, Fasano CA, Papapetrou EP, Tomishima M, Sadelain M, Studer L (2009): Highly efficient neural conversion of human ES and iPS cells by dual inhibition of SMAD signaling. *Nat Biotechnol*. 27:275-280.

10. Shi Y, Kirwan P, Smith J, Robinson HP, Livesey FJ (2012): Human cerebral cortex development from pluripotent stem cells to functional excitatory synapses. *Nat Neurosci*. 15:477-486, S471.

11. Andersen CL, Jensen JL, Orntoft TF (2004): Normalization of real-time quantitative reverse transcription-PCR data: a model-based variance estimation approach to identify genes suited for normalization, applied to bladder and colon cancer data sets. *Cancer research*. 64:5245-5250.

12. Untergasser A, Cutcutache I, Koressaar T, Ye J, Faircloth BC, Remm M, et al. (2012): Primer3--new capabilities and interfaces. *Nucleic Acids Res*. 40:e115.

13. Livak KJ, Schmittgen TD (2001): Analysis of relative gene expression data using real-time quantitative PCR and the 2(-Delta Delta C(T)) Method. *Methods (San Diego, Calif)*. 25:402-408.

14. Schneider CA, Rasband WS, Eliceiri KW (2012): NIH Image to ImageJ: 25 years of image analysis. *Nat Methods*. 9:671-675.

15. Srivastava DP, Woolfrey KM, Penzes P (2011): Analysis of dendritic spine morphology in cultured CNS neurons. *J Vis Exp*.e2794.

16. Du P, Kibbe WA, Lin SM (2008): lumi: a pipeline for processing Illumina microarray. *Bioinformatics*. 24:1547-1548.

17. Warde-Farley D, Donaldson SL, Comes O, Zuberi K, Badrawi R, Chao P, et al. (2010): The GeneMANIA prediction server: biological network integration for gene prioritization and predicting gene function. *Nucleic Acids Res*. 38:W214-220.

18. Duffy JB (2002): GAL4 system in Drosophila: a fly geneticist's Swiss army knife. *Genesis*. 34:1-15.

19. Truman JW, Bate M (1988): Spatial and temporal patterns of neurogenesis in the central nervous system of Drosophila melanogaster. *Dev Biol*. 125:145-157.

20. Gargano JW, Martin I, Bhandari P, Grotewiel MS (2005): Rapid iterative negative geotaxis (RING): a new method for assessing age-related locomotor decline in Drosophila. *Exp Gerontol*. 40:386-395.

21. Zeisel A, Munoz-Manchado AB, Codeluppi S, Lonnerberg P, La Manno G, Jureus A, et al. (2015): Brain structure. Cell types in the mouse cortex and hippocampus revealed by single-cell RNA-seq. *Science*. 347:1138-1142.

22. Mostafavi S, Ray D, Warde-Farley D, Grouios C, Morris Q (2008): GeneMANIA: a real-time multiple association network integration algorithm for predicting gene function. *Genome Biol*. 9 Suppl 1:S4.

23. Kim HJ, Kim NC, Wang YD, Scarborough EA, Moore J, Diaz Z, et al. (2013): Mutations in prion-like domains in hnRNPA2B1 and hnRNPA1 cause multisystem proteinopathy and ALS. *Nature*. 495:467-473.

24. Jung S, Chung Y, Oh YJ (2017): Breaking down autophagy and the Ubiquitin Proteasome System. *Parkinsonism Relat Disord*.

25. Molochnikov L, Rabey JM, Dobronevsky E, Bonucelli U, Ceravolo R, Frosini D, et al. (2012): A molecular signature in blood identifies early Parkinson's disease. *Mol Neurodegener*. 7:26.

26. Ramkumar A, Murthy D, Raja DA, Singh A, Krishnan A, Khanna S, et al. (2017): Classical autophagy proteins LC3B and ATG4B facilitate melanosome movement on cytoskeletal tracks. *Autophagy*. 13:1331-1347.
